# Cardiac Progenitor Cells from Stem Cells: Learning from Genetics and Biomaterials

**DOI:** 10.3390/cells8121536

**Published:** 2019-11-28

**Authors:** Sara Barreto, Leonie Hamel, Teresa Schiatti, Ying Yang, Vinoj George

**Affiliations:** 1 Guy Hilton Research Centre, School of Pharmacy & Bioengineering, Keele University, Staffordshire ST4 7QB, UK; s.barreto-francisco@keele.ac.uk (S.B.); teresa.schiatti@gmail.com (T.S.); y.yang@keele.ac.uk (Y.Y.); 2 RCSI Bahrain, P.O. Box 15503, Adliya, Bahrain; 18211372@rcsi-mub.com

**Keywords:** cardiac progenitor cells, induced pluripotent stem cells, transdifferentiation, direct reprogramming, genetic engineering, cardiac tissue engineering, biomaterials

## Abstract

Cardiac Progenitor Cells (CPCs) show great potential as a cell resource for restoring cardiac function in patients affected by heart disease or heart failure. CPCs are proliferative and committed to cardiac fate, capable of generating cells of all the cardiac lineages. These cells offer a significant shift in paradigm over the use of human induced pluripotent stem cell (iPSC)-derived cardiomyocytes owing to the latter’s inability to recapitulate mature features of a native myocardium, limiting their translational applications. The iPSCs and direct reprogramming of somatic cells have been attempted to produce CPCs and, in this process, a variety of chemical and/or genetic factors have been evaluated for their ability to generate, expand, and maintain CPCs in vitro. However, the precise stoichiometry and spatiotemporal activity of these factors and the genetic interplay during embryonic CPC development remain challenging to reproduce in culture, in terms of efficiency, numbers, and translational potential. Recent advances in biomaterials to mimic the native cardiac microenvironment have shown promise to influence CPC regenerative functions, while being capable of integrating with host tissue. This review highlights recent developments and limitations in the generation and use of CPCs from stem cells, and the trends that influence the direction of research to promote better application of CPCs.

## 1. Cardiac Regeneration—A Problem to Solve or A Solution with Promise?

With morbidity rates associated with cardiovascular diseases in the decline in the developed world from improved treatments and pharmacological intervention, scientists and clinicians have been approaching therapies recently for these diseases with vigor. However, there is still no reliable therapy for acute cardiac conditions like myocardial infarction (MI), which account for nearly half of all cardiovascular deaths in the industrialized world [1,2]. Regenerative medicine-based strategies for infarcted myocardium have shown promise in preclinical animal models as well as early clinical trials [3]. Whilst these have demonstrated some physiological improvements in ventricular function, they were associated with very low cell retention after some weeks, suggesting a paracrine effect of transplanted cells rather than functional integration within the damaged tissue [4].

The heart was long viewed as a post-mitotic or terminally differentiated organ with no ability to regenerate or repair, a dogma that has been challenged abundantly in recent years [5,6]. Cardiac regeneration, following injury, is still an unresolved debate over whether it is attributed to dedifferentiation and proliferation of resident cardiomyocytes or from an inherent trigger in differentiation of cardiac stem or progenitor cells in putative cell niches within the heart [7,8,9,10,11]. The turnover of cardiomyocytes in the adult heart is around 1% per year which is insufficient to counter the loss caused by MI that can lead to loss of around 1 billion cardiomyocytes [12]. Therefore, the only long-term solution relies on heart transplantation, but this does not come without its own issues such as insufficient number of donors coupled with the requirement for a life-long immunosuppressive therapy. This catapulted research towards cell-based therapies for cardiac regeneration [13]. Cardiomyocytes are the main cardiac cell type that is lost in cardiovascular disorders, like heart failure, myocardial infarction, and ischemia, and therefore, replacing these cells could potentially restore heart function. However, transplanting cardiomyocytes to repair diseased hearts has shown to yield only transient responses as most cells are eventually lost in the host environment [14,15]. This is because cardiomyocytes have very limited proliferative ability and as a result, they are unable to repopulate and replenish the damaged tissue efficiently [16,17]. Furthermore, other cell types like smooth muscle cells, and endothelial cells can suffer from collateral damage and their functional renewal is vital for effective heart regeneration [18]. This puts emphasis on the role of a precursor cell type capable of extensive expansion and differentiation into the key cell players of cardiac regeneration.

Even though some level of cell turnover has been observed in the adult heart, cells with self-renewal or potency capabilities are generally considered lacking in this tissue [19]. Nevertheless, several studies report the evidence of a progenitor population from resident cardiac stem cells (CSCs) in the heart, called Cardiac Progenitor Cells (CPCs) [20,21,22,23]. In contrast to terminally differentiated cardiomyocytes, CPCs are highly proliferative and can theoretically differentiate into all the necessary cardiac cell types for effective reconstitution of damaged cardiac tissue and promoting its neovascularization [14,18,20,21,24,25,26,27,28,29,30,31]. Therefore, CPCs present a more effective cell source than cardiomyocytes for cell-based regenerative strategies. However, the application of CPCs has not been straight-forward particularly in chronic infarcts, where CPCs are associated with senescence, decreased telomerase activity and increased apoptosis [7]. Cell therapy using CPCs generally involve transplantation of in vitro-expanded CPC populations which in turn yield mild improvements in cardiac function [32]. However, long term prognosis with such therapies are poor owing to reduced cell viability and inefficient engraftment into the host tissue. This is compounded by the somewhat hostile microenvironment created by MI, from scar formation and associated inflammatory or tissue alterations, which compromises the effectiveness of such therapies [33,34,35]. There are also reports that the administration of CPCs predisposed the risk of cardiac arrhythmias and teratoma formation [36]. Therefore, better understanding of the CPC cell behavior in dynamic pathophysiological microenvironments could aid in developing strategies to optimize their contribution to cardiac repair.

Various approaches have been developed to generate CPCs ex vivo, in the hope of obtaining reliable source of cells that can trigger mechanisms of cardiac regeneration. For example, CPCs from the heart tissue (also known as putative CPCs) can be isolated and expanded in vitro [27,37,38,39,40]. However, such cells are hard to access and are present in low numbers in the tissue, making them extremely challenging to harvest and realize their potential [41]. Pluripotent stem cells, such as embryonic stem cells (ESCs) and induced pluripotent stem cells (iPSCs), are thought to be a superior alternative cell source since they could potentially provide an unlimited supply of cardiac progenitor cells. However, ESC-based therapy faces several challenges like immunogenicity, high risk of tumor formation and the characteristic ethical concerns, which have prevented their clinical application [42,43]. On the other hand, iPSCs avoid the ethical issues associated with ESCs and allows for the development of patient-specific derived CPCs, which represents an advantage over other cell sources in the creation of immune-compatible cardiac therapies [44,45]. However, with issues surrounding the safety of iPSC-based therapies, in terms of the potential risk of tumor formation associated with such therapies or immune rejection of iPS-derived cells from a common donor, scientists are looking at reprogramming from a different perspective [46,47,48]. Reprogramming patient somatic cells into other cell types, bypassing the step of stem cell generation, can potentially overcome issues with translating iPSC technology. This process is known as direct cellular reprogramming or transdifferentiation, and might represent a more robust approach to rapidly generate sufficient numbers of CPCs from somatic cells for therapy [49].

**Figure 1:**
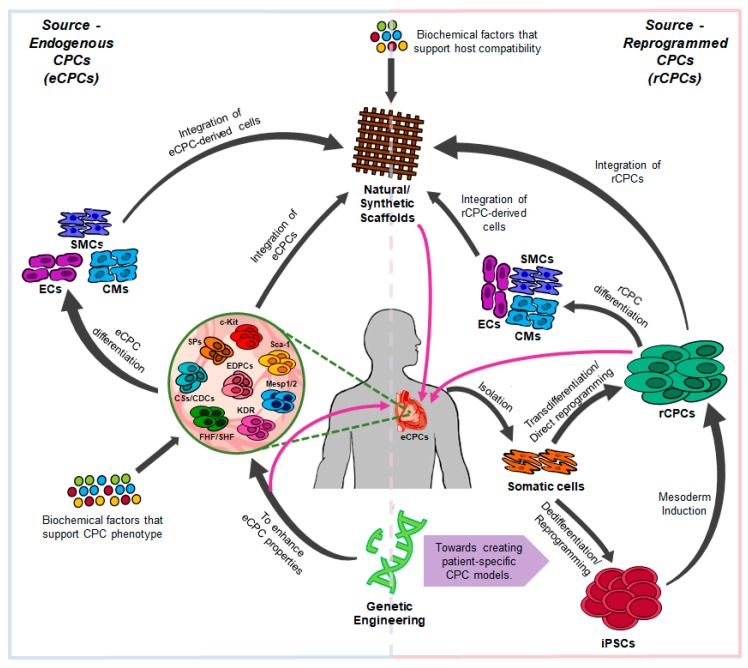
The interplay between genetics and biomaterials for understanding Cardiac Progenitor Cells (CPCs) biology, function, and its regenerative applications. eCPCs (endogenous CPCs), rCPCs (reprogrammed CPCs), iPSCs (induced Pluripotent Stem Cells), SPs (Side Population-derived CPCs), CSs/CDCs (Cardiospheres/Cardiosphere-Derived Cells), EDPCs (Epicardium-derived CPCs), FHF/SHF (First Heart Field-/Second Heart Field-derived CPCs) CMs (Cardiomyocytes), SMCs (Smooth Muscle Cells), ECs (Endothelial Cells).

This review focuses on the ongoing progress and limitations in generating CPCs from iPSCs and through direct reprogramming. It will start by providing a concise introduction about the various cardiac progenitor cells identified in embryonic and adult heart tissues. The review will then move towards discussing reprogramming approaches that were successful in generating CPCs and the functionality of these CPC-derived cells. Strategies to improve efficiencies of current protocols and tissue engineering advances to mimic CPC microenvironment and in vivo applications of CPCs will also be evaluated. Finally, the review will finish with a summary of existing challenges and limitations and future directions for CPC research, hopefully convincing readers it is a promising strategy for cardiac regeneration (Figure 1).

## 2. Cardiac Progenitor Cells (CPCs) In Vivo

Progenitor cells are distinct from embryonic stem cells as they have a predetermined differentiation fate and therefore, their ability to self-renew and differentiate into other cell types is restricted [19]. CPCs generate cells of the three cardiac lineages: cardiomyocytes, smooth muscle cells and endothelial cells. These cells are also responsible for the maintenance of cardiac homeostasis under physiological and pathological conditions [50]. Several studies have identified and isolated multiple CPC populations from distinct stages of cardiac development and heart locations. These cells are collectively characterized according to their cell surface and genetic marker expression profiles. The various CPCs reported to date are described below and their specific features are summarized in Table 1.

### 2.1. c-KIT-Expressing CPCs

The first-ever detected CPCs were isolated from female rats and were characterized by the expression of the stem cell surface marker c-KIT [28]. These CPCs are present throughout the ventricular and atrial myocardium, particularly in the atria and the ventricular apex [28]. These progenitor cells also express the cardiac transcription factors NKX2.5, GATA4, and MEF2C, and are negative for hematopoietic lineage markers, such as CD45, CD34, CD3, CD14, CD16, CD19, CD20 and CD56 [50,51,52]. They are self-renewing, clonogenic and are able to differentiate into the three cardiac cell types in vitro and in vivo [28,53]. The c-KIT receptor binds to the Stem Cell Factor (SCF) which leads to the activation of the signaling pathways Phosphoinositide 3-kinase/Protein Kinase B (PI3K/AKT) and p38 Mitogen-Activated Protein Kinase (MAPK) [54,55]. These pathways regulate a variety of CPC functions like self-renewal, proliferation, survival, and migration [54,55,56,57]. Even though c-KIT CPCs contribute to the generation of cardiomyocytes at earlier stages of embryonic development and right after birth, this ability is mostly lost in the adult heart and very low percentages of new cardiomyocytes seem to originate from these CPCs [58,59,60]. Therefore, the improvement of cardiac function by c-KIT CPCs might be a result of paracrine factors rather than the production of de novo cardiomyocytes [58,61]. Furthermore, c-KIT expression is considered necessary but not sufficient to define CPCs [62].

### 2.2. SCA1-Expressing CPCs

Another CPC population present in adult hearts expresses the Stem Cell Antigen 1 (SCA1). The cells were first identified in adult mouse hearts [11] and are predominantly located in the atrium, the intra-atrial septum, the atrium-ventricular boundary and scattered within the epicardial layer [37]. SCA1 is a cell surface protein of the Ly6 gene family and it was initially used to isolate hematopoietic stem cells [63]. Additionally, SCA1 is widely expressed by stem and progenitor cells from a variety of tissues, including the heart, and it has roles in cell survival, proliferation and differentiation [63]. Several studies have shown that SCA1 CPCs are negative for hematopoietic lineage markers and are able to differentiate into the three cardiac lineages [11,64]. These CPCs also have the ability of homing in response to injury and contribute to neovascularization in vivo [11,65,66]. Although this CPC population seems promising for cardiac regeneration, their translational relevance is not without caveats. First, all the SCA1 CPC populations identified to date display different gene expression profiles and distinct differentiation potential [37,66,67,68,69,70,71]. In addition, several studies have shown that the benefits resulted from the transplantation of these CPCs might be predominantly due to paracrine mechanisms as these cells differentiate into cardiomyocytes with very low efficiency [66,68,70,71]. Finally, SCA1 is only present in murine cells and a human ortholog of SCA1 has yet to be identified [63]. Therefore, the nature of the epitope target of SCA1 in humans and the nature of regeneration of the associated CPC population have yet to be determined.

### 2.3. MESP1/2-Expressing CPCs

During the development of mesoderm, embryonic cells express the transcription factor Mesoderm Posterior Protein 1/2 (MESP1/2), which is essential for proper cell migration [15,72,73,74]. MESP1/2 expression marks the first step in the commitment of the nascent mesoderm into the myocardial lineages, and it describes the first population of multipotent cardiac progenitor cells that produce the various cardiac cell types of the heart [72,75]. Although MESP1/2 CPCs show increased cardiac potential, in comparison to other CPC types, they are not irreversibly committed to the cardiac fate [76]. Consequently, there is a possibility that these cells will differentiate into derivates of the paraxial mesoderm and skeletal muscle [77,78]. Furthermore, MESP1/2 is only transiently expressed during embryonic development, which increases the difficulty of tracking the expansion and differentiation of the CPCs [79,80].

### 2.4. KDR/FLK1-Expressing CPCs

During cell movement from the primitive streak to the anterior regions of the embryo, the precardiac mesodermal cells start to express a receptor for Vascular Endothelial Growth Factor (VEGF) called KDR/FLK1 [20,81]. These FLK1-expressing progenitor cells have the ability to generate cells from both hematopoietic and cardiac lineages [20,81,82,83]. Selection between these two lineages is determined by the levels of FLK1 activity [81]. For example, high expression of FLK1 promotes differentiation towards hematopoietic lineages, whereas low or absent FLK1 expression stimulates cells to follow the cardiac fate [81,82]. These negative FLK1-expressing cells further generate a second FLK1^+^ cell population that represents the first multipotent cardiac progenitor cells that are permanently committed to the cardiogenic fate [20,29]. Because KDR/FLK1 displays a broad expression, it is frequently used in combination with other cardiac markers, such as Platelet-Derived Growth Factor-alpha (PDGFRα), C-X-C chemokine Receptor type 4 (CXCR4) and sometimes MESP1/2, to enrich for CPCs [79,80].

### 2.5. CPCs from the First and Second Heart Fields

The cardiac mesoderm contains two unique progenitor cell pools that give rise to the primary and secondary heart fields [20,22]. The two fields develop sequentially and display distinctive molecular profiles that lead to the formation of different heart components. CPCs of the first heart field (FHF) express the transcription factor NKX2.5, whereas CPCs from the second heart field (SHF) express the transcription factor ISL1 [15,22,40,75,84]. FHF-derived CPCs are more difficult to isolate owing to a lack of unique markers except for NKX2.5 [84]. The hyperpolarisation-activated nucleotide-gated cation channel HCN4 has been suggested as an additional marker for FHF, however, this marker might isolate a more restricted CPC that preferentially generates cells of the conduction system [85,86,87]. Regardless of the markers, FHF-CPCs predominantly differentiate into cardiomyocytes and have some tendency towards smooth muscle lineages [21]. On the other hand, ISL1 CPCs can generate cells of all the three cardiac lineages and they are responsible for producing most of the cardiomyocytes (around 40%) during heart development [22,30,40]. In addition, these CPCs have also been identified in the adult heart, specifically in the outflow tract, atria and right ventricle [30,40].

### 2.6. Epicardium-Derived CPCs

Several studies have demonstrated that a specific CPC population present in the postnatal and adult heart is derived from the epicardium. They express the transcription factor Wilms tumor 1 (WT1) and are originally derived from CPCs of the second heart field [88]. Additionally, these CPCs emerge from epicardial cells that migrate into the myocardium and undergo epithelial-to-mesenchymal transition (EMT) [88,89]. The epicardial-derived CPCs can differentiate into several different cell types such as coronary smooth muscle cells, cardiomyocytes, endothelial cells, perivascular and cardiac interstitial fibroblasts, albeit with varying efficiencies [51,88,89,90,91,92,93]. Even though WT1 CPCs could potentially be an additional cell source for cardiac regeneration, these cells seem to share some characteristics with c-KIT CPCs: they participate in cardiomyocyte formation during cardiac development but are present at extremely low levels in the adult heart [9,88,90,92]. Stimulatory factors like peptide thymosin beta 4 (Tβ4) can potentially reactivate the developmental program of adult epicardial cells, however, the reactivated cells still exhibit distinct phenotype from their embryonic counterparts, raising doubts about their cardiogenic potential [92].

### 2.7. Side Population-Derived CPCs

Side populations (SPs) have been detected in various tissues, including the heart, and are enriched for stem and progenitor cell activity [38,94,95,96,97,98]. These cells are generally identified in vitro by their ability to export the DNA Hoechst dye from their nuclei when stained [94,95]. This dye efflux is performed by an ATP (Adenosine Triphosphate)-binding cassette transporter (also known as ABC transporter protein) that is present in their cellular membranes [94,95]. The phenomenon causes the side population cells to contain much lower concentrations of the dye in their nuclei compared to other cell types, allowing for their isolation using cell sorting techniques [95]. The main ABC transporter protein used to identify cardiac SPs is the ABCG2, which was demonstrated to have a role in stem cell proliferation and differentiation and is expressed in SP cells during early development and in the postnatal heart [38,94,95,99]. These cardiac SPs can be found in the perivascular and interstitial areas of the heart, and display self-renewal, homing and multipotency [94,97,100,101,102]. Noseda et al. (2015) demonstrated that cardiac SPs, co-expressing SCA1 and PDGFRα, displayed high clonogenicity and multilineage potential [103]. They particurlaly demonstrated that clones derived from cardiac SPs subjected to long-term propagation (more than 10 months and 300 doublings) still resembled freshly isolated SP cells, showing maintainance of phenotype, self-renewal and tri-lineage capacity and absence of replicative senescence. However, cardiac SPs exhibit a few disadvantages that could potentially prevent their clinical application. For instance, the differentiation potential of human SPs has not been thoroughly investigated [38]. In addition, the multipotency of SPs might be attributed to their heterogeneous nature as they are composed of several subpopulations with distinct differentiation potential (cardiac, hematopoietic and mesenchymal) [38,104]. Therefore, it is still inconclusive on which markers can predict the SP subpopulation with the best cardiac potential.

### 2.8. Cardiosphere-Derived CPCs

Cardiospheres contain a mixture of stromal, mesenchymal and progenitor cells that are isolated from human heart biopsy cultures [39,52]. They represent a niche-like environment containing a mixture of cells, with cardiac-committed cells in the center and supporting cells, such as mesenchymal and endothelial progenitor cells, in the periphery of the spherical cluster [105,106]. Many cells can be harvested from these cell clusters and they are called cardiosphere-derived cells (CDCs) [52,105]. However, like in the case of c-KIT and epicardial CPCs, the regenerative potential of CDCs is questionable as it has been shown that cardiac repair by these cells mainly results from paracrine mechanisms rather than cell generation [105]. 

## 3. Generation of CPCs from Human iPSCs

Native CPCs are present in very low numbers in the heart tissue, and therefore, a larger source of cells is required for efficient cardiac regeneration [41]. The reprogramming of human adult somatic cells into embryonic stem cell-like cells (known as iPSCs) using defined factors opened new possibilities for the generation of patient-specific pluripotent cells. In turn, human iPSCs could potentially offer an unlimited source of differentiated cells and in the process, offer the chance to recreate the development process of CPCs in vitro [121]. This section will provide a detailed description and assessment of current methods used to induce, expand and maintain CPCs derived from iPSCs.

Several techniques have been developed to modulate cardiac differentiation in iPSCs (Table 2). However, the efficiencies for cardiac differentiation can vary considerably between iPSC lines [121,122,123,124]. Regardless of the type of culture employed, the first step in all protocols involves the dedifferentiation of a chosen cell type into a pluripotent state using conventional reprogramming factors, such as OCT4, SOX2, KFL4 and c-MYC [44,45]. Once pluripotency has been achieved, the following step is to induce cardiac differentiation of the iPSCs. Different methods have been employed to accomplish differentiation of iPSCs into cardiomyocytes: embryoid body (EB); monolayer-based cultures supplemented with growth factors, serum or small molecules, matrices, and co-culture with visceral endodermal stromal (END2) layers [15,122,125]. Recent protocols utilise a monolayer culture with a serum-free medium, such as mTeSR1 or E8 medium, which maintains iPSC pluripotency and self-renewal in a feeder-free culture [126,127]. Unfortunately, these studies predominantly focused on the generation of iPSC-cardiomyocytes and not necessarily the homogeneity of CPC population entering the cardiac lineages.

In addition to the nature of the pluripotent culture employed, the type and timing of growth factors and/or small molecules added throughout the protocol affects cardiomyocyte differentiation efficiency. Early differentiation protocols only employed growth factors that modulate key signaling pathways involved in cardiomyogenesis, like Bone Morphogenic Protein (BMP), Activin/Nodal and Fibroblast Growth Factor (FGF) signaling pathways [15,128]. Such factors included Activin A, BMP2/4 and FGF2 which induce cardiac mesoderm formation in iPSCs [15,29,122,128,129]. Lian et al. (2012) demonstrated that iPSC differentiation towards cardiac lineages could be accomplished by exclusively using small molecule modulators of the Wingless (WNT) signaling pathway [130]. Minami et al. (2012) also showed that combining analogous WNT modulators during the early and middle stages of the cardiac differentiation process can further enhance the protocol’s efficiency [131]. Many protocols rely on adding a Glycogen Synthase Kinase (GSK3) inhibitor, such as CHIR99021 (CHIR), to the medium for 24 h to activate the canonical WNT signaling [126,130,132]. Induction of the canonical WNT signaling stimulates the expression of the mesoderm marker Brachyury (T) in undifferenced iPSCs, initiating mesoderm induction [126,132]. Once T^+^ cells have been established, the WNT signaling is then suppressed to direct the mesodermal cells towards the cardiac fate [126]. Several inhibitory molecules can be used, like XAV939, inhibitor of WNT production (IWP), inhibitor of WNT response (IWR) or an exogenous β-catenin small hairpin RNA (shRNA). After 3/4 days of WNT signaling suppression, iPSC-derived T^+^ mesodermal cells begin to express cardiac transcription factors, like NKX2.5, ISL1, FLK1, and PDGFRα, which transitions into the CPC population.

More recent studies have been successful in generating CPCs from iPSCs using a single small molecule, potentially reducing costs, time and labor. For instance, the immunosuppressant cyclosporin-A (CSA) was shown to stimulate differentiation of FLK1-positive mesodermal cells into FLK1^+^/CXCR4^+^/VE-cadherin^−^ CPCs and cardiomyocytes [133,134]. When CSA was added to the medium, the CPC and cardiomyocyte yield was 10 to 20 times higher compared to untreated cells. Additionally, the generated cardiomyocytes exhibited molecular, structural and functional properties of adult cardiomyocytes. However, additional factors and/or other protocols may be required to produce cells from the other cardiac lineages as FLK1^+^/CXCR4^+^/VE-cadherin^−^ CPCs have an exceptionally low endothelial potential and cannot differentiate into smooth muscle cells [110,133]. Furthermore, the study used co-culture with END2 cells to induce cardiac differentiation in iPSCs, which prevents reproducibility of the protocol due to the presence of END2-derived growth factors at unknown concentrations [135]. Another study also demonstrated that treating human iPSCs with the cardiogenic small molecule isoxazole (ISX-9) for 7 days stimulated the expression of CPC markers [136]. These CPCs expressed NKX2.5, GATA4, ISL1, and MEF2C and were able to generate cardiomyocytes, smooth muscle cells and endothelial cells under basal differentiation conditions. Furthermore, ISX-9 seems to modulate key signaling pathways involved in cardiomyogenesis, like VEGF, Activin A and canonical and non-canonical WNT signaling. The study also demonstrated that the small molecule might participate in CPC generation by upregulating activators involved in both canonical and non-canonical WNT pathways in a temporal and sequential manner (WNT3A at day 3 and WNT5A and WNT11 at day 7, respectively).

Therefore, the application of iPSC technology in CPC research has great prospects for improving current cardiac regeneration approaches through the development of novel cell therapies, disease models and drug screens. However, most studies using iPSCs in cardiac regeneration predominantly focus on producing cardiomyocytes and improving their maturation [15,126,130,137,138,139,140,141,142]. Current knowledge about associating this with the generation of iPSC-CPCs, however, remain limited.

## 4. Direct Reprogramming into CPCs

The discovery of iPSC reprogramming prompted studies to evaluate if it would be possible to reprogram somatic cells directly into other cell types without an iPSC intermediate stage, a process known as transdifferentiation or direct reprogramming. Transdifferentiation has shown to be a much quicker process than dedifferentiation into iPSCs, with the former taking only a few days to achieve, whereas the latter can last up to 3 weeks plus differentiation time to produce the desired cell lineages. With the added advantage of avoiding potential cumulative mutation or epigenetic changes, generally associated during complex iPSC reprogramming processes, direct reprogramming of somatic cells can potentially offer a simpler, faster and safer alternative to generate cells compared to iPSC dedifferentiation [41]. Most transdifferentiation studies in the cardiac field involve the generation of fully differentiated cardiac cells, particularly cardiomyocytes, rather than cardiac progenitor cells [159,160,161,162,163,164,165,166,167,168,169,170,171,172]. Potentially, using transdifferentiation protocols to generate CPCs might be a superior approach for regenerative medicine applications. This section focuses on the current approaches that are associated with producing CPCs from direct reprogramming.

### 4.1. Partial Somatic Cell Reprogramming into CPCs

Some studies have developed transdifferentiation protocols that involve a transient stage of pluripotency of somatic cells before they continue into CPC fates. The use of reprogramming factors (OCT4, SOX2, KLF4 and C-MYC) seems to be enough to initiate resetting of epigenetic memory of somatic cells towards a stem cell path (partial reprogramming), but the factors alone are insufficient to directly activate cardiac lineage-specific genes for directed differentiation [159]. In order to achieve lineage commitment, signaling molecules involved in cardiogenesis, like BMPs, WNT modulators and FGFs, need to be activated in the cultures [14,159], similar to differentiation protocols for cardiomyocytes from iPSCs. One study demonstrated that secondary mouse embryonic fibroblasts can be converted into CPCs using a technique developed by Wang et al. (2014) called Cell Activation and Signaling-Directed (CASD) lineage conversion [165], which combines reprogramming and cardiac-specific factors to induce cell activation and direct cell fate towards cardiogenesis, respectively [14]. Zhang et al. (2016) transiently exposed the mouse fibroblasts to reprogramming medium containing doxycycline and JAK inhibitor 1 (JI1) for 6 days, and then to transdifferentiation medium with CHIR99021 and JI1 for 2 days to induce cardiac differentiation. Following this, the cells are treated with a mixture of CHIR99021, BMP4, Activin A, and SU5402 (inhibitor of FGF, VEGF, and PDGF signaling) for 3 days. The obtained CPCs from this protocol expressed the proliferative marker Ki-67, the typical cardiac transcription factors GATA4, MEF2C, TBX5 and NKX2.5, and the cell surface molecules FLK1 and PDGFRα and were capable of producing cells from the three cardiac lineages. Efe et al. (2011) also demonstrated that transient expression of pluripotent markers (OCT4, SOX2, KLF4 and C-MYC) followed by exposure to chemically defined media containing BMP4 and the JAK inhibitor JI1 induced cardiac conversion of mouse embryonic and tail-tip fibroblasts [159]. JI1 was added to the reprogramming media for 9 days and from day 9, BMP4 was added and the media was subsequently changed to RPMI supplemented with N2 and B27 lacking vitamin A for 5 additional days. This protocol upregulated the expression of several CPC markers such as NKX2.5, GATA4, and FLK1 by day 9/10.

Wang et al. (2014) were able to significantly reduce the number of reprogramming factors to successfully stimulate cardiac transformation in mouse fibroblasts [165]. This protocol involved a single transcription factor (OCT4) and a cocktail of small molecules: an activin A/TGF-β receptor (ALK4/5/7) inhibitor (SB431542), GSK inhibitor (CHIR), Lysine (K)-Specific Demethylase 1 (LSD1/KDM1) inhibitor (parnate/tranylcypromine) and an adenylyl cyclase activator (forskolin). Mouse fibroblasts were first exposed to the reprogramming media, containing the small molecules, for 15 days. This was followed by media change to RPMI supplemented with N2 and B27 lacking vitamin A and addition of BMP4 during the first 5 days. CPC markers, like FLK1, MESP1, ISL1, GATA4, and Ki-67, can be detected around days 15–20. These cells went on to differentiate into cardiomyocytes, endothelial cells and smooth muscle cells under specific conditions. Another study developed an entirely chemical reprogramming protocol that utilised a larger combination of small molecules compared to Wang et al. (2014): CHIR, the ALK5 inhibitor RepSox, forskolin, the histone deacetylase (HDAC) 1 inhibitor valproic acid (VPA), parnate and the retinoid pathway activator TTNPB [173]. Mouse fibroblasts were exposed to the reprogramming cocktail for 16 days and CPC markers could be detected around day 8-20. The markers identified included SCA1, ABCG2, WT1, FLK1, and MESP1, demonstrating that the protocol can generate CPC populations. Most of the studies described protocols predominantly focused on their ability to generate cardiomyocytes from somatic cells using some iPS factors, and whilst CPCs were observed in some of these studies, their characteristics were not necessarily a focus of their attention and would warrant some investigation in their potency independently.

### 4.2. Direct Somatic Reprogramming into CPCs

Direct reprogramming of somatic cells involves the transdifferentiation into other cell types without an iPSC intermediate stage. One study showed that CPCs can be directly generated from adult mouse fibroblasts from different tissues (cardiac, lung and tail tip) using a 11- (MESP1, MESP2, GATA4, GATA6, BAF60C, SRF, ISL1, NKX2.5, IRX4, TBX5 and TBX20) or a 5- Factor (MEF2C, TBX5, GATA4 NKX2.5, BAF60C) reprogramming protocol [24]. Both protocols led to the formation of CPCs expressing the genes *NKX2.5*, *MEF2C*, *MESP1*, *TBX20*, *IRX4*, and the cell surface protein CXCR4, independently of factor combination and tissue origin of the fibroblasts. The CPCs also showed downregulation of fibroblasts-specific genes, such as *FSP1*, and could differentiate into the three cardiac lineages. Furthermore, adding a canonical WNT activator, and a JAK/STAT activator during the reprogramming process can increase the protocol efficiency, leading to the production of more CPCs. Even though the 11-factor and 5-factor protocols generated CPCs with comparable characteristics, they differ in the amount of CPC colonies generated, with the former producing more, and in the expression of smooth muscle cell and endothelial cell markers in CPC-differentiated cells, with the 5-factor protocol-based CPCs generating more of these markers than the 11-factor system. Another study showed that human dermal fibroblasts can be directly reprogrammed into CPCs by overexpressing the genes *MESP1* and *ETS2* [174]. In this specific reprogramming protocol, human dermal fibroblasts are converted into CPCs through a 4-day co-expression of ETS2 and MESP1 using lentiviral vectors, which is then followed by Activin A and BMP2 treatment for another 2 days. Human ETS2 is a transcription factor involved in development, apoptosis and oncogenic transformation and when co-expressed with MESP1, induces the expression of BMP2, initiates the Activin A/Nodal signaling and stimulates the emergence of CD31/PECAM-1 (endothelial cells) and KDR cells (CPCs). ETS2 could potentially be substituted by other ETS transcripts, such as ETS1, FLI1, ETV1, ETV5, ERG and ETV that are also highly abundant in the developing heart, and might function similarly to ETS2 in reprogramming human somatic cells into CPCs.

All these protocols described required the use of viral vectors, usually lentiviruses, to deliver the reprogramming factors into cells. This implied host cell genome changes which could potentially affect its suitability for translational applications. One method that addresses this concern is through the delivery of reprogramming proteins, related to transcription factors, directly into cells. These proteins can modulate the gene expression of cells to convert them into other cell types. For example, using a nonviral-based protein delivery system with the cardiac transcription factors GATA4, HAND2, MEF2C, and TBX5 induces reprogramming of human dermal fibroblasts into CPCs [41]. Additionally, adding growth factors such as BMP4, Activin A and basic Fibroblast Growth Factor (bFGF) can further stimulate and sustain potency towards a CPC state. This combination increased the cellular expression of CPC markers (FLK1 and ISL1) and decreased the expression of fibroblast-specific markers (COL1A2 and FSP1). Furthermore, the protocol demonstrated high efficiency in direct transdifferentiation, converting more than 80% of the human dermal fibroblasts into CPCs.

### 4.3. Somatic Reprogramming into Cardiospheres

Recent studies have shown that adult skin fibroblasts from mouse and human can be converted into cardiospheres that, in turn, have the potential to generate CPCs [175,176]. For this, the skin cells were first reprogrammed with the Yamanaka factors SOX2, KLF4 and OCT4 overnight, followed by media change to Knockout Serum Replacement-based media for 18 days and finally treatment with the GSK3 inhibitor BIO and Oncostatin for 2 days [175,176]. The resulted cardiospheres resembled endogenous cardiospheres formed from the cellular outgrowth of cardiac explants in vitro [39], but produced a higher number of MESP1, ISL1-, and NKX2.5- expressing cells [175,176]. On passaging, the cardiospheres became enriched with CPCs expressing c-KIT, FLK1 and CXCR4, which were able to differentiate into cardiomyocytes [175]. However, human cardiospheres do not display spontaneous beating and fail to propagate in vitro compared to mouse cardiopsheres, suggesting different signaling pathways being utilized for somatic reprogramming into cardiospheres in both mice and humans [175,176].

### 4.4. In Vivo Direct Reprogramming

One exciting potential of direct reprogramming is its application *in vivo*, in which endogenous cardiac cells would be directly converted into CPCs to repair the damaged myocardium. This approach could represent an improvement in promoting cardiac regeneration as it bypasses the several issues associated with cellular transplantation [166,177]. In addition, it avoids the need for cell harvesting, expansion, maintenance, and/or effective delivery systems, which are current challenges faced by cellular in vitro methods. In vivo direct reprogramming takes advantage of the heart native environment that might contain extracellular matrix proteins and growth factors that could make cells more permissive for functional reprogramming and lead to the formation of more mature cardiac cells [160,177,178,179,180]. In a study using an in vivo zebrafish model [181], cardiac ventricular injury induced the expression of Notch and RALDH2 in atrial cardiomyocytes, which caused the cells to lose their sarcomeric organization and re-express CPC transcription factors, such as GATA4, HAND2, NKX2.5, TBX5, TBX20 and MEF2. Once these dedifferentiated atrial cardiomyocytes reached the ventricle, they further expressed ventricle-specific markers, like Iroquois Homeobox Protein Ziro 1 (IRX1A) and ventricular Myosin Heavy Chain (vMHC), and differentiated into ventricular cardiomyocytes. Another study demonstrated that adult murine atrial and ventricular cardiomyocytes can acquire properties of CPCs through spontaneous dedifferentiation in vitro [182]. The dedifferentiated cardiomyocytes gave rise to CPCs that expressed the cardiac markers c-KIT, GATA4, and NKX2.5, self-organised into cardiospheres and were able to differentiate into functional cardiomyocytes and endothelial cells [182]. These results were further investigated by Zhang et al. (2015) in vivo using a MI mouse model [183]. They specifically analysed DNA methylome changes during cardiomyocyte dedifferentiation and observed that cardiomyocyte-specific genes, like Myosin Light Chain Kinase 3 (*MYLK3*) and Myosin Heavy Chain 6 and 7 (*MYH6* and *MYH7*), became hypermethylated (repressed), whereas cell cycle and proliferation genes, such as Epiregulin (*EREG*) and SRY-box 4 (*SOX4*), were hypomethylated (upregulated) in the generated CPCs. This concept could potentially be applied in in vivo CPC reprogramming. However, the molecular mechanisms involved in somatic cell dedifferentiation are not fully elucidated and more information is needed to identify the factors responsible.

Although in vivo reprogramming shows great potential, it has only been employed to derive fully differentiated cardiac cells, specifically cardiomyocytes, and not CPCs as such [160,177,178,179,180,184,185]. Therefore, even though direct reprogramming seems to be a suitable approach to generate CPCs, there are still some issues that influence its application in regenerative therapeutics. These include sub-optimal efficiencies in transdifferentiation protocols for CPC generation and lack of in-depth characteristics of CPC potency, differentiation potential and functionality of their derivatives.

## 5. In Vitro Culture of CPCs Derived Through Reprogramming Protocols

Establishing reprogramming protocols to generate CPCs from iPSCs and somatic cells is essential to advance CPC research for cardiac regeneration. However, the field also faces issues regarding the isolation, propagation, and expansion of CPCs in vitro. This section focuses on the current methods that have been successful in isolating, expanding and maintaining CPCs in vitro.

### 5.1. Isolation of CPCs

Isolation of CPCs is usually performed based on their characteristic gene expression patterns and surface markers (see Table 1). For example, ISL1 and NKX2.5 genes are frequently used to identify CPCs [186]. However, these genes are transiently expressed in cells which can lead to the isolation of a heterogeneous cell population containing various CPCs with distinct self-renewal and differentiation potential [186]. When using only cell surface markers, a combination of at least two markers is frequently used as a single surface marker seems insufficient to discriminate a CPC signature. For instance, Nsair et al. (2012) demonstrated that the co-expression of two cell surface markers, FLT1 (VEGFR1) and FLT4 (VEGFR3) specifically identifies ISL1/NKX2.5-expressing CPCs [187]. This combination was also shown to be more effective in identifying homogenous CPC populations (approximately 89% pure) compared to other combinations, such as FLK1 alone or FLK1 with PDGFRα. Furthermore, the isolated CPCs were able to differentiate into the three cardiac lineages and engraft into the host tissue. One study by Nelson et al. (2008) used the cell surface markers CXCR4 and FLK1 to isolate a more restricted CPC from a heterogeneous FLK1 positive population [188]. Zhou et al. (2017) also demonstrated that the marker SIX2 is able to target temporally distinct cell subpopulations from second heart field-associated CPCs [189]. One very recent study (Torán et al., 2019) used proteomic and genomic approaches to comprehensively characterize the proteome of human adult c-KIT CPCs [190]. It was demonstrated that these CPCs highly express 4 surface markers: GPR4 (G protein-coupled receptor 4), CACNG7 (calcium voltage-gated channel auxiliary subunit gamma 7), CDH5 (VE-cadherin) and F11R (F11 receptor) in comparison to mesenchymal stem cells, human dermal fibroblasts and cardiac fibroblasts. More research, however, will be required to further clarify the role of these proteins in CPC functions.

Thus, new markers are continuously being discovered to isolate specific CPC populations. However, they are frequently identified in CPCs derived from neonatal/adult tissue but fewer in ESC-CPCs and iPSC-CPCs [107,133,134,190,191,192]. Further validation of such markers is vital to assign a common signature that accurately identifies these cells.

### 5.2. Expansion and Maintenance of iPSC-CPCs

The maintenance of β-catenin concentration seems to be an efficient method for CPC expansion in vitro [187,193]. Applying GSK3 inhibitors, like WNT3A, CHIR, or 6-bromoindirubin-3′-oxime/BIO, can promote CPC expansion and suppress myocytic differentiation, leading to the formation of a relatively homogenous CPC colony [193]. Furthermore, the combination of a WNT/β-catenin inhibitor (IQ-1) and a ROCK inhibitor (Thiazovivn) is also able to expand CPCs in a feeder-free medium for a minimum of 4 weeks, while maintaining their multipotent state (more than 90% remained multipotent) [187]. IQ-1 is a selective β-catenin inhibitor that targets the signaling mediated by the protein’s interaction with p300. This suppresses p300 pro-differentiation function and stimulates a pluripotency state. Furthermore, WNT signaling seems to interact with other signaling pathways, such as Notch and FGF signaling, to stimulate the expansion of CPCs [194,195]. For example, activation of the Notch signaling by Notch1 represses expansion, self-renewal and β-catenin activity in CPCs [195]. Activation of both WNT and FGF signaling pathways enhances ISL1 CPCs in a cooperative manner [194]. Therefore, using biomolecules that inhibit and activate the Notch and FGF signaling, respectively, together with WNT activators might facilitate CPC expansion. Notably, inhibition of FGF signaling has also been demonstrated to enhance CPC expansion, but this inhibition is suggested to affect only a subset of CPCs (expressing SCA1) [196] and therefore, warrants further investigation.

Several studies have shown that persistent inhibition of the BMP signaling enhances expansion of CPCs and prevents their differentiation [186,197,198]. For example, the BMP inhibitor Gremlin 2 (GREM2), whose expression initiates in NKX2.5^+^ CPCs after cardiac mesoderm specification and follows cardiac lineage differentiation, promotes proliferation of CPCs from iPSCs by suppressing the BMP4 receptor activity [197]. This effect was demonstrated to be consistent across distinct iPSCs lines and independent of the differentiation method used. However, GREM2 is also able to induce differentiation of CPCs into the cardiac cell subtypes. Therefore, timing and potency of this BMP antagonist may need careful evaluation to CPCs and avoid spontaneous differentiation. Notably, GREM2 appears to only increase the number of KDR^low^ and NKX2.5^+^ CPCs in vitro, and its function seems to be lost in the adult heart. Ao et al. (2012) used a second-generation BMP inhibitor called Dorsomorphin homologue 1 (DMH1) that was able to enrich CPCs, expressing Branchyury, MESP1 and ISL1 markers, from pluripotent cells [198]. Additionally, DMH1 was shown to be a more selective inhibitor of BMP type 1 receptors compared with other BMP inhibitors. This selective inhibition is, therefore, best applied during early stages of cardiac differentiation (pre-mesoderm and cardiac mesoderm stages) in order to increase the number of CPCs.

Another molecule that enhances CPC expansion in vitro is Ascorbic acid (AA) [143]. AA was shown to enhance the expansion of isolated iPSC-derived FLK1^+^/CXCR4^+^ CPCs through the MEK-ERK1/2 pathway by promoting collagen synthesis. However, the effects of AA on other CPC types need to be evaluated before AA can be used as a universal factor for efficient CPC expansion. Birket et al. (2015) used a cocktail of molecules modulators of the FGF, VEGF, PDGF, BMP, Nodal, AKT and hedgehog signaling pathways (SU5402, DMH1, SB431542, Insulin-Like Growth Factor 1 (IGF1) and Smoothened Agonist (SAG)) that was capable of expanding CPCs for more than 40 population doublings [186]. However, this study used MYC-transduced iPSC lines and consequently, the method needs further assessment using CPCs derived from non-transgenic iPSCs. Bao and colleagues (2017) developed two protocols, with and without serum, to maintain self-renewal and stimulate expansion of human iPSC-derived epicardial CPCs for long periods of time [152,153]. Both methods involve the addition of a TGF-β inhibitor, such as SB431542 or A83-01, to the medium. The epicardial CPCs can either be cultured in LaSR basal medium, which contains albumin, or in RPMI with ascorbic acid and insulin (RPMI/Vc/Ins), a xeno-free/chemically defined medium. Cells kept in LaSR basal medium can be maintained for up to 2 months, whereas CPCs in RPMI/Vc/Ins can be cultured for approximately 24 days before they start undergoing epithelial-to-mesenchymal transition (EMT) and lose their morphology. The use of a gentler dissociating buffer (Versene) also seemed to improve expansion efficiency of the CPCs from human pluripotent stem cells (iPSCs and ESCs) after 8 passages [152]. One study developed a Good Manufacturing Practice (GMP)-compatible system for the expansion of CPCs, using stirred tank bioreactors and microcarrier technology [199]. Human CPCs from three different donors were inoculated with microcarriers (Cytodex 1 coated with CELLstart^TM^CTS^TM^) for up to 7 days. The microcarrier-based stirred cultures lead to a cell suspension increase of 3-fold and greater cell viabilities compared with standard static T-flask monolayers. Furthermore, the CPCs in the culture system expressed the markers CD44, CD105, CD166, KDR, GATA4, and TBX5. This method provides tight control of environmental cues to mimic physiological conditions, which could potentially improve the production of high-quality CPCs for therapeutic applications.

### 5.3. Expansion and Maintenance of Transdifferentiated CPCs

CPCs derived from direct reprogramming of somatic cells seem to have similar requirements as iPSCs-CPCs for expansion and maintenance. For instance, adding a canonical WNT activator and a JAK/STAT activator to the cultures was shown to maintain the proliferative and multipotent state of the CPCs for several passages (over 20 and 30 passages for a 5- and 11- Factor reprogramming protocol, respectively) without continuous expression of the reprogramming factors [24]. However, CPC maintenance and expansion potential can be negatively affected when utilising somatic cells from tissues other than cardiac tissue, like lung and skin tissues. Furthermore, fibroblast-derived CPCs can be alternatively expanded and maintained using a combination of signaling molecules (BMP4, Activin A, CHIR, and SU5402) that synergistically repress cardiac differentiation and sustain CPC self-renewal [14]. In this case, the CPCs’ undifferentiated morphology, gene expression pattern and cell surface molecule expression remain the same for more than 18 passages regardless of the tissue origin of the donor cells.

Overall, the requirements for in vitro culture of CPCs involved the precise temporal activation and suppression of several signaling pathways. It remains challenging to expand CPCs while maintaining their self-renewal and multipotent differentiation potential as the process is extremely complex, preventing the development of standard conditions yet. This can be more complicated when considering CPCs derived from iPSCs and direct reprogramming and their associated characteristics [186,200,201,202]. Therefore, more comparative studies of current protocols will be imperative to establish standard in vitro culture conditions that are optimal for the isolation, expansion and maintenance of specific CPCs.

## 6. Strategies to Improve CPC Reprogramming

Strategies for producing CPCs are still developing with time. Whilst the concept of CPC generation through reprogramming or transdifferentiation has taken precedence to produce desired cardiac lineages, the protocols suffer from poor efficiency or lack of mechanistic insight to achieve the target population and desired functional improvement. Strategies to accelerate proliferation and extend replicative lifespan of CPCs are being essentially employed to understand and potentially overcome the inherent limitations of patient CPC populations derived from compromised, aged, or damaged myocardium. With developments in genetic engineering approaches and factors, such as CRISPR gene editing, epigenetic modulators and/or microRNAs, and its significance in cardiac development, there seems scope for applying this in the field of CPC regeneration and address some of the current limitations. This section will describe examples of such strategies in the context of CPCs.

### 6.1. Genetic Engineering with PIM1

Genetic engineering with PIM1, has been applied in CPCs to enhance their properties, like proliferation, survival and differentiation [203]. Pro-viral insertion site for the moloney murine leukemia virus (*PIM1*), a proto-oncogene serine/threonine-protein kinase, is highly expressed in bone marrow, tumor cells and fetal heart and is associated with many signaling pathways, mostly related to anti-cell apoptosis and cell cycle regulation [204]. Mohsin and colleagues (2013) genetically modified patient-derived human CPCs (hCPCs) with PIM1 kinase (termed hCPCeP) to increase proliferation, telomere length, survival and decrease expression of cellular senescence markers, rejuvenating the phenotypic and functional properties of hCPCs, in an effort to ameliorate the cumulative effects of age and disease [205]. The PIM1-engineered cells also showed increased commitment to the three cardiac lineages [203]. Interestingly, the effect of PIM1 in hCPCeP normalizes after several rounds of passaging, consistent with the notion that PIM-1 can transiently increase mitosis coupled with telomere stability (increased TERT activity) and without resultant oncogenic transformation through a c-MYC synergy. These properties of hCPCeP can be modulated by targeted localization of PIM1 in mitochondrial or nuclear components, conferring an optimal stem cell trait irrespective of patient-associated cell heterogeneity [206]. Furthermore, intramyocardial injection of hCPCeP into cardiomyopathic challenged-SCID mice demonstrate increased cellular engraftment and differentiation with improved vasculature and reduced infarct size [203]. Similar results were also observed when using murine CPCs [207] but these earlier studies relied largely on viral delivery methods to induce PIM1 overexpression. In an alternative strategy, a non-viral modified plasmid-minicircle (MC) was used as a vehicle to deliver PIM1 into mouse CPCs (mCPCs) in vitro and the myocardium in vivo [208]. Mice with PIM1-MC injection showed increased protection compared to control groups measured by ejection fraction at 3- and 7-days post injury, supporting the potential of a non-cell based therapeutic approach for treatment of ischemic heart disease and MI.

### 6.2. CRISPR in Context with CPCs

In an effort to identify previously unknown regulators of cardiomyocyte differentiation from human ESCs (hESCs) through quantitative proteomics, Murry lab [209] demonstrated that DAB2 (Disabled 2) plays a functional role in cardiac lineage specification towards cardiomyocytes by being preferentially upregulated in CPCs. CRISPR/Cas9 deletion of Dab2 in zebrafish embryos was used to show increase in WNT/β-catenin signaling and consequent decrease in cardiomyocyte number, suggesting that inhibiting WNT/β-catenin signaling by DAB2 (or analogous inhibitors like the Dickkopf WNT signaling Pathway Inhibitor 1 (DKK1)) can be crucial in maintaining cardiomyocyte numbers from CPCs in the developing heart. Supporting this mechanism, the same lab, using antisense knockdown and CRISPR/Cas9 mutagenesis in hESCs and zebrafish, went on to demonstrate that Alpha Protein Kinase 2 (ALPK2) is temporally expressed during specification of CPCs (but not in endocardial-like endothelial cells), and cardiac commitment through negative regulation of WNT/β-catenin signaling [210]. In a more recent study [211], CRISPR-mediated ablation of *Furin* gene in mouse CPCs, whose product is a natural target of *Nkx2.5* repression during heart development, produces abnormalities in embryo characterized by reduced proliferation of CPCs and their premature differentiation, suggesting *Furin* mediates some aspects of *Nkx2.5* function in heart and is necessary for CPC differentiation. This role of *Furin* in the maturation of CPCs is, in part, mediated by the modulation of the BMP pathway by *Nkx2.5*. Therefore, genetic engineering using CRISPR has been pivotal in recent years to identify mechanisms associated with CPCs and continue to show promise with a perpetual trend in CRISPR advances.

### 6.3. Epigenetic Modulators

Distinct cell types display different epigenetic profiles that leads to differential gene expression. Cellular reprogramming is associated with changes in the epigenetic signature of cells. During these epigenetic transitions, proteins called epigenetic modulators bind to specific regions of the chromatin and regulate the transcription of genes. Therefore, inhibition and/or overexpression of these modulators might affect cardiac reprogramming efficiency [41,212]. For example, knockdown of the polycomb ring finger pro-oncogene *Bmi1* in several fibroblast types (murine embryonic, neonatal and adult tip tail and adult cardiac fibroblasts) results in the activation of core cardiac transcription factors, such as GATA4, ISL1 and TBX20, which converts the cells into cardiomyocytes [212]. Additionally, Zhou et al. (2016) demonstrated that silencing of *Bmi1* allowed for efficient cardiomyocyte reprogramming using just two factors (MEF2C and TBX5). The induced cardiomyocytes displayed features of advanced maturity, such as contractile activity, sarcomere structures and periodic calcium oscillation. Therefore, it would be useful to investigate the role of *Bmi1* in the context of CPC reprogramming, considering the significance of ISL1 upregulation under *Bmi1* depletion. Another epigenetic modulator that could potentially be employed in CPC reprogramming is the BAF chromatin remodeling protein BAF60A. BAF60A is thought to have a role in the maintenance of CPC self-renewal thought interaction with TBX1 [213,214]. TBX1 seems to recruit BAF60A onto the promoter region of *WNT5A* gene, upregulating its expression in CPCs [214]. WNT5A is a non-canonical WNT pathway ligand that is highly expressed in CPCs derived from the SHF, and it cooperates with another non-canonical WNT ligand, called WNT11, to induce development of CPCs from the two heart fields [215]. Accurate identification of the cellular epigenetic barriers could potentially reduce the number of reprogramming factors employed to generate CPCs and ultimately, lead to faster and safer protocols.

### 6.4. MicroRNAs

MicroRNAs are short non-coding RNA molecules that bind to messenger RNA and repress gene expression. MicroRNAs show a promising alternative to traditional reprogramming protocols as they are easily delivered and display low toxicity in animal models [184]. In addition, several microRNA transcripts can be packed into a single delivery vector, which could potentially increase reprogramming efficiency. However, most studies have mainly examined the use of microRNAs in converting somatic cells directly into cardiomyocytes, not CPCs as such [180,184,216]. Nevertheless, microRNAs have been shown to modulate CPC functions [217,218,219] (see Table 3). Sirish et al. (2012) investigated the miRNA expression changes in CPC development [219]. They identified 8 differentially expressed microRNAs (miR-103, -130a, -17, -130b, -208b, -185, -200b and -486) in mouse neonatal and adult LIN^−^/c-KIT^+^ CPCs. The target proteins of microRNAs were predicted to be predominantly involved in cell proliferation, with a few proteins having roles in cell organisation, development, metabolic process, adhesion, homeostasis, activation, communication, and motility. The group also demonstrated that overexpression of the miR-17-92 cluster, which targets cell cycle proteins, in adult CPCs increased their proliferative capacity by 2-fold in vivo. Two studies showed that the microRNAs miR-1, -499 and -204 repress proliferation and stimulate differentiation in committed SCA1^+^ CPCs [217,218]. Additionally, Xiao et al. (2012) revealed that inhibition of miR-204 suppressed CPC differentiation and promoted proliferation without affecting cell viability [218]. A study in 2016 identified several microRNAs that regulate cardiac fate, like let-7, miR-18, miR-302 and the miR-17-92 cluster, in MESP1^+^ CPCs [220]. It was also shown that the CPCs were particularly enriched for the miR-322/-503 cluster which targets the CUG-binding protein Elav-like family member 1 (CELF1). Ectopic CELF1 expression promoted neural lineage-specification at the expense of cardiomyocyte differentiation in the CPCs. Therefore, miR-322/-503 may be a key regulator in promoting the cardiac program in early mesodermal cells by cross-suppressing other lineages. Garate et al. (2018) investigated the expression of microRNAs during the differentiation of human pluripotent stem cells (hPSCs) towards mesoderm and cardiac cells [221]. They found several microRNA families (miR-302, C19MC, miR-17/92 and miR-26) that were highly expressed in EpCAM/CD326-negative and NCAM/CD56-positive mesoendodermal progenitor cells (MPCs) [222]. The microRNA families identified were speculated to be associated with the epithelial to mesenchymal transition occurring during the development of mesoderm. However, the specific roles of the microRNAs in CPCs will need to be determined as MPCs are able to generate all the mesoendodermal lineages, including cardiovascular, hematoendothelial and mesenchymal. One very recent study by Cheng et al. (2019) showed that the ischemic heart secretes microRNAs (miR-1a, miR-133a, miR-208a and miR-499) that mobilised LIN^−^/c-KIT^+^ bone marrow progenitor cells (BM PCs) into the site of injury, where they proliferated and promoted vascularisation [223]. These results demonstrated the principle of employing microRNAs to target endogenous progenitor cells to enhance ischemic cardiovascular repair. Therefore, as molecular mechanisms regulated by microRNAs during CPC development get explored more, they offer a suitable choice of target for improving CPC generation from iPSCs or for transdifferentiation. 

## 7. Tissue Engineering with CPCs and CPC-Derived Cardiomyocytes

Several studies have demonstrated that the cells generated from CPCs, particularly cardiomyocytes, display an immature phenotype similar to that of embryonic cardiac cells [3]. However, when the CPCs are transplanted into a host environment, the differentiated cells reach a more advanced maturity, such as greater organisation of sarcomeres and formation of gap junctions (in the case of cardiomyocytes) and development of tubular-like structures (for smooth muscle and endothelial cells) [11,24,27,28]. Furthermore, CPCs seem to have distinct differentiation potential in vitro and in vivo [96,111]. This could mean that the microenvironment of the heart might have a key role in CPC functions. Stem and progenitor cells reside in specific tissue microenvironments, called niches, which provide protection and support to the cells [241]. A way to potentially enhance CPC regenerative potential could be to mimic their microenvironment. Cardiac tissue engineering aims to achieve this goal by combining multiple microenvironment components, such as cells, extracellular matrix (ECM) and biochemical factors like BMP2, VEGF, bFGF, DKK1, and IGF1, to create cardiac tissue constructs. Therefore, determining the ideal matrix for supporting CPCs and their derivatives is paramount. In principle, the scaffold matrix should be biodegradable, immune-privileged, provide electrical and/or mechanical properties for cell coupling and assembly, and support vascularisation [242,243]. Two types of materials are typically employed in the production of scaffolds for tissue engineering: natural matrices and synthetic matrices. This section will describe different types of scaffolds that have been used in combination with CPCs and CPC-derived cardiomyocytes (Table 4).

### 7.1. Natural Scaffolds

Natural matrices have the advantage of being composed of native ECM cues that modulate cell behavior [243,244]. These scaffolds can comprise pure ECM elements, like hydrogels made from natural materials such as fibrin, alginate, gelatin, and collagen, or acellular tissue which displays the biochemical and biomechanical properties (tensile strength and composition) of the native ECM tissue [245,246]. Three independent studies used a fibrin patch seeded with CPCs (murine and human) to develop a tissue construct, which was then tested in vivo [247,248,249]. Vallée et al. (2012) specifically utilized BMP2-primed murine ESCs seeded onto fibrin matrices as single cells, small cluster and embryoid bodies [249]. These constructs were then engrafted onto myocardial infarcted rat hearts, which led to a reduction in remodeling and deterioration of cardiac functions. Seeded cells were identified by the expression of the cardiac genes MESP1, NKX2.5, MEF2C, TBX6 and GATA4, speculating a CPC-related population. The transplanted cells were also able to colonize the outer connective tissue where they differentiated into cardiomyocytes and promoted neovascularization. The results from Vallée et al. (2012) encouraged two other studies to apply their tissue engineering approach with human CPCs [247,248]. Bellamy et al. (2015) and Menasché et al. (2015) seeded human CPCs, expressing the markers SSEA1 and ISL1, in a fibrinogen patch [247,248]. The two studies differed in the number of CPCs used, Bellamy et al. (2015) used 700,000 cells whereas Menasché et al. (2015) used 4 million cells; and in the in vivo model chosen, myocardial infarction rats and a 68-year-old patient suffering from severe heart failure, respectively. Improvement of contractility and attenuation of ventricular remodeling was observed in both studies. It was also shown that these benefits were likely a result of paracrine factors secreted by the transplanted CPCs rather than de novo generation of tissue. Gaetani and colleagues (2012 and 2015) used 3D printing with SCA1^+^/CD105^+^ fetal CPCs, which are referred to as human fetal cardiomyocyte progenitor cells (hCMPCs), and three types of natural scaffolds (pure, RGD-modified alginate and a hyaluronic acid/gelatin-based matrix) [250,251]. The hCMPCs were able to migrate from the scaffolds, colonize the surrounding areas and form tubular-like structures [250,251]. Another study by Christoforou et al. (2013) used murine iPSC-derived CPCs mixed within a fibrin/Matrigel hydrogel that were applied in polydimethylsiloxane (PDMS) molds and cultured for 14 days in vitro [157]. These CPCs expressed NKX2.5, GATA4, c-KIT and either FLK1 or SCA1 and differentiated into mature cardiomyocytes that aligned into unidirectional myofilament and displayed abundant electromechanical connections. This study also concluded that accessibility to oxygen and nutrients within tissue constructs greatly affects integration of the implanted cells.

Native ECM generally comprise of various components such as glycosaminoglycans (GAGs), collagen, fibrinogen, hyaluronic acid and hydroxyapatite (HA) [246]. To mimic this, recent studies have applied natural scaffolds generated from the decellularisation of tissues. This technique removes any cells present in the tissue while preserving its original 3D architecture and ECM. Two studies have combined decellularised scaffolds with iPSC-derived CPCs [252,253]. Lu et al. (2013) used human iPSC-CPCs that were positive (low) for the marker KDR and negative for c-KIT to repopulate a whole decellularised mouse heart [252]. The CPCs differentiated into cardiomyocytes, endothelial cells and smooth muscle cells, and efficiency to a specific lineage could be changed with the addition of growth factors. The recellularised scaffolds displayed vessel-like structures, spontaneous contraction, uniform wave propagation in some regions, and the ECM seemed to stimulate proliferation of CPCs and formation of wider myofilaments of cardiomyocytes. However, drawbacks of this study included the uneven recellularisation of the heart constructs which led to weaker mechanical forces and incomplete synchronization, and inability to generate cells of the conduction system and cardiac fibroblasts. Although natural scaffolds retain the ultrastructure and biological information of the native tissue, there is a risk of immunological reaction, disease transmission (in case of animal-derived materials) and are generally variable in their physical properties [243,245].

### 7.2. Synthetic Scaffolds

The ideal synthetic scaffold should be biocompatible, degradable, display a surface that allows for cell attachment, migration and differentiation, and a macrostructure that supports cell growth and nutrient and waste exchange [245]. Structure and properties of synthetic scaffolds, like the associated mechanics, chemistry and degradation rate, can be easily customised for the type of cells being used [243,245,246]. Two studies employed self-assembling peptide nanofibres with CPCs and tested the constructs in vivo [254,255]. Both studies used two distinct experimental designs: Padin-Iruegas et al. (2009) seeded adult rat Lin^−^ c-KIT^+^ CPCs onto nanofibres tethered with IGF1, whereas Tokunaga et al. (2010) used adult mouse SCA1^+^ CPCs mixed with Puramatrix^®^ (3D Matrix, Ltd.) (no tethered factors). The CPCs in Tokunaga et al. (2010) nanofibres minimally contributed to de novo cardiomyocyte generation and had no differentiation potential towards endothelial lineages [255]. The benefits observed were associated to effects from paracrine signaling. On the other hand, Padin-Iruegas et al. (2009) showed that continued IGF1 release from nanofibres enhanced CPC survival and proliferation, and stimulated differentiation into cardiomyocytes, smooth muscle cells and endothelial cells [254]. Additionally, the regenerated cardiomyocytes were able to couple with resident cardiomyocytes, and the smooth muscle cells and endothelial cells formed vascular structures. These studies demonstrated that functionalising self-assembling peptide nanofibres can potentially support long-term CPC survival, proliferation and differentiation, and lead to a more robust maturity of the CPC-derived cells, especially if applied in the human CPC context. Li et al. (2011) used a solution made of mouse cardiosphere-derived cells and degradable poly(N-isopropylacrylamide) hydrogel and performed in vitro testing of the effects of scaffold stiffness and presence/absence of collagen on the cells’ functions [256]. The hydrogels with medium stiffness and collagen were optimal for cardiosphere-derived cells differentiation into cardiomyocytes, which displayed the highest expression of maturation genes (MYH6 and cTNT). Unfortunately, there were no reports on the effects of the hydrogels on cardiosphere-derived cells differentiation potential towards smooth muscle cells and endothelial cells. Liu et al. (2015) also employed nanofibres with CPCs, but they used poly(l-lactic acid) and mouse ESC-derived CPCs [257]. These CPCs were positive for ISL1 and GATA4 and differentiated into the three cardiac lineages in both in vitro and in vivo conditions. Additionally, differentiation potential towards endothelial lineages was improved in vivo compared to that of in vitro. The scaffolds supported CPC survival, engraftment, proliferation and integration with the host tissue, and stimulated the expression of intercellular coupling proteins (connexin 43) and maturation of cardiomyocytes.

One study used a novel concept called “scaffold-in-scaffold” to promote human CPC growth and differentiation in vitro [258]. The aim of this approach was to create a structure with different physical characteristics to better mimic the ECM microarchitecture. The multitexture 3D scaffold was composed of a polyethylene glycol diacrylate (PEGDa) woodpile and a softer PEGDa hydrogel. Human LIN^−^ SCA1^+^ CPCs seeded on these scaffolds highly differentiated into cardiomyocytes, which aligned in an orderly manner. However, robust cardiomyocyte maturation, such as sarcomeric organisation and formation of gap junctions, was not achieved. In addition, there were no reports on the differentiation potential towards other cardiac lineages.

Synthetic biomaterials are a great promise to constructing 3D microenvironments with adjustable features. However, they still come with a few limitations, such as poor biocompatibility, incomplete polymer degradation, and some toxicity, that will need to be addressed systematically to achieve better cellular responses.

**Figure 2:**
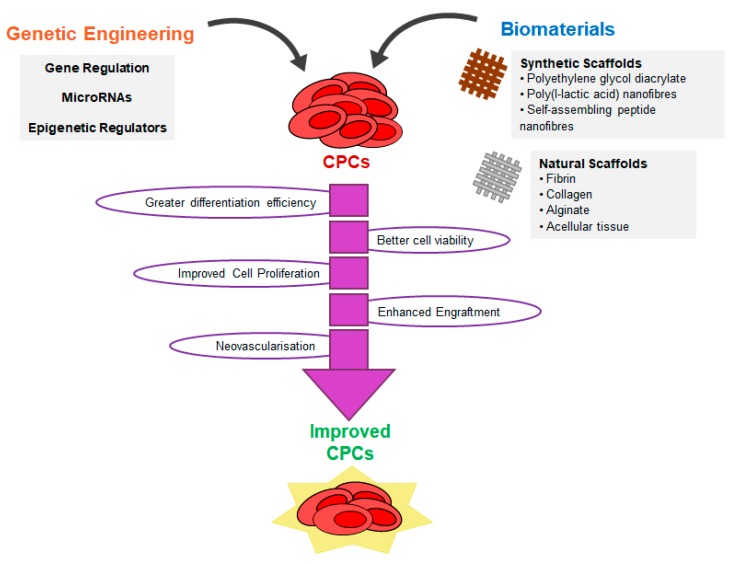
Promising strategies to improve CPC characteristics and functionality. Strategies for producing CPCs to date through reprogramming or transdifferentiation has been associated with poor efficiency or lack of mechanistic insight to achieve the target population and desired functional improvement. With a range of tools for genetic engineering or gene modulation, and with advances in tissue engineering approaches, new strategies have been applied in this field to accelerate proliferation, enhance differentiation, extend replicative lifespan or improve functionality or engraftment of CPCs (detailed in Section 6 and Section 7).

A significant trend that has been popular with human Pluripotent Stem Cell-Cardiomyocytes (hPSC-CMs), has been the implementation of electrically-compatible scaffolds or biomaterials (in 2D or 3D) compatible with standard electrophysiology measurements to stimulate hPSC-CM electrical behavior and consequently its mature electrophysiological phenotypes (see Table 5). This would be a strategy for exploration with CPCs as we improve our understanding of the CPC niche. Furthermore, while most of the studies described above employed ESC-derived or putative CPCs on scaffolds, studies using patient-specific CPCs from iPSCs or from transdifferentiation in engineered scaffolds to model phenotypes are very rare. Therefore, with potential improvements in cardiac tissue engineering and mechanistic understanding of responses in situ, the CPC niche can be exploited to assess normal and disease-associated cardiac cell behavior to produce better regenerative outcomes (Figure 2).

## 8. In Vivo Applications of Human CPCs

The end-goal of in vitro and animal in vivo studies in CPC research is to provide enough evidence regarding the efficacy and safety of cell therapies for further application in human trials. This is not without the caveat that, despite promising results from in vitro and animal models, the translation to clinical trials still suffer from serious inefficiencies in desirable outcomes over long term, costing billions of dollars in the process [280]. Even though there is not yet an agreement on the CPC population that displays the best regenerative capacity, a variety of CPCs have been used or are being used in clinical trials, which are summarized in Table 6.

The first-ever clinical trial using CPCs, called SCIPIO (Stem Cell Infusion in Patients with Ischemic cardiOmyopathy) used human LIN^−^ c-KIT^+^ CPCs to improve postinfarction left ventricular dysfunction. However, this study has now been retracted due to concerns about the randomisation and lack of integrity of certain data [281,282]. In 2012, the randomised phase I trial CADUCEUS (CArdiosphere-Derived aUtologous stem CELLs to reverse ventricUlar dySfunction) employed cardiosphere-derived cells to reduce scarring after myocardial infarction [283]. These cells were obtained from endomyocardial biopsy specimens and were transplanted into patients 1.5–3 months post-myocardial infarction using intracoronary infusion. The results showed that the cells led to an improvement in viable heart tissue and a reduction of scarring. Differentiation potential of cardiosphere-derived cells towards cardiac lineages remained to be elucidated and thus, it is likely that the benefits observed in the CADUCEUS study were a result of paracrine factors. In the same year, another phase I trial called ALCADIA (AutoLogous human CArdiac-Derived stem cell to treat Ischemic cArdiomyopathy) used autologous human CPCs in combination with a controlled released of bFGF in patients suffering from ischemic cardiomyopathy and heart failure [284,285]. These CPCs expressed the mesenchymal surface markers CD105 and CD90 and were also derived from endomyocardial biopsy specimens. The cells were injected intramyocardially and a biodegradable gelatin hydrogel sheet containing bFGF was then implanted on the epicardium, which covered the injection sites areas. However, as in the case of the CADUCEUS study, the benefits observed, such as attenuation of adverse ventricular remodelling and neovascularisation, were probably due to paracrine mechanisms as there was no compelling evidence that the employed CPCs can differentiate into cardiomyocytes in vivo [284,286]. A more recent trial published in 2018, named ESCORT (Transplantation of Human Embryonic Stem Cell-derived Progenitors in Severe Heart Failure), used hESC-derived CPCs, expressing the markers SSEA1/CD15 and ISL1, embedded in a fibrin gel [287]. The scaffold was then delivered onto the epicardium of the infarct area. The aim of the study was to confirm the safety and feasibility of the therapy rather than evaluating its regenerative effects in the patients. Further investigation will be needed to thoroughly assess the benefits of the fibrin gel patch in severe heart failure.

There are also reports on phase I and II clinical trials assessing the use of autologous cardiosphere-derived cells in paediatric patients suffering from hypoplastic left heart syndrome [288,289]. The phase I TICAP (Transcoronary Infusion of CArdiac Progenitor cells in patients with single ventricle physiology) demonstrated that the approach was safe and feasible for improving cardiac function after 18 months [288]. The safety of the therapy was also analysed at 36 months post-transplantation [290]. There was no tumour formation and the initial observed benefits were enhanced, with attenuation of ventricular stiffness and improvement of ventriculoarterial coupling. The results obtained from TICAP were further confirmed by the phase II PERSEUS (Cardiac Progenitor Cell Infusion to Treat Univentricular Heart Disease) [289]. Furthermore, the therapy is currently being tested in a phase III trial (APOLLON) [291] and applied in paediatric patients diagnosed with dilated cardiomyopathy (phase I trial TICAP-DCM: Transcoronary Infusion of CArdiac Progenitor cells in paediatric Dilated CardioMyopathy) [292], for which results are still waiting.

Most trials involving CPCs come with limitations in employing small sample sizes or lack of blinded assessment, which ultimately leads to inconclusive results regarding the therapies’ efficiency in recovering from cardiac disorders. In addition, it is still inconclusive whether the positive results are attributed to intracoronary infusion of CPCs themselves or from paracrine factors as speculated by some trials. It will, therefore, be imperative to perform future clinical trials with a broader assessment of study subjects and an established human reproducible model to better explore the CPCs’ regenerative capacity in human hearts.

## 9. Current Challenges and Limitations

There is still a lot of debate on the effect that CPCs play a role in cardiac regeneration and repair in the context of diseases like MI, demonstrating increased left ventricular ejection fraction, decreased infarct size, and an increase in hemodynamic function following infusion of autologous CPCs. Even though there is a growing emphasis on the application of CPCs for cardiac regeneration, its impact is still obscure, particularly owing to its heterogeneous nature and mechanistic silencing from deep-rooted complexities associated with the nature of the cardiomyopathic disease. For example, there is still no consensus regarding which CPC population is the ideal cell type for cell-based regenerative therapies and which combination of markers accurately characterise CPCs. Additionally, the characteristic epigenetic, gene, protein and secretome profiles of most CPCs remain unclear [19,41]. This could elucidate how phenotypes and genotypes of CPCs alter throughout their development and their effects on self-renewal and potency potentials. Furthermore, not many studies have investigated and compared the therapeutic efficacy of different CPCs. The ideal CPC type should be able to tolerate autologous transplantation, expand extensively in vitro, differentiate into mature cardiac cell subtypes and integrate with the host cells [299].

Viral transduction remains the main approach applied in most reprogramming processes (both in vitro and in vivo) as it shows the greatest efficiency. However, this is associated with a risk of genome integration and activation of oncogenic genes. In addition, the currently developed protocols require the use of both reprogramming and growth factors which substantially increases their complexity and final cost. It is, therefore, imperative to develop a more effective and simpler gene transfer methods that ensure cell therapies are safe and display a good cost-benefit ratio.

Furthermore, the populations of CPC-derived cells are heterogeneous and frequently represent immature cells, which could potentially lead to arrhythmias, lower long-term stability and poor integration when transplanted [3,300]. The mechanisms involved in cardiac lineage subtype specification will need to be fully investigated and optimised to produce purer and more mature populations of the desired cell types from the CPCs. With the growing pace of CRISPR strategies and its potential to address limitations associated with genetic control and regulation, it will not be surprising that this will be applied to CPCs for this purpose in the very near future.

Epigenetic profiles seem to strongly affect reprogramming efficiencies for both iPSCs and transdifferentiation technologies. For example, using cells from non-cardiac tissue organs or aged tissue negatively affects the cardiogenesis capability of iPSCs [301]. The success of reprogramming a cell fate relies on the ability to overcome the several epigenetic barriers present in somatic cells. The more distinct the donor somatic cells are from the cardiac tissue, the higher the number of epigenetic barriers that need to be overcome and consequently, the harder it is to reprogram the cells. Therefore, understanding the epigenetic regulatory mechanisms involved CPC formation might be vital to improving reprogramming efficiency.

Another limitation in CPC research is that many studies have been performed in rodent models, which display distinct cardiac anatomy and physiology from the human heart. Additionally, current techniques developed using animal cells will need to be further validated for human context. For example, the direct reprogramming protocol involving the three core cardiac genes GATA4, MEF2C and TBX5 (also known as GMT) was demonstrated to induce mouse fibroblasts into cardiac cells, but it was insufficient to convert human fibroblasts [164].

For future preclinical trials, the relationship between the number of CPCs and their effects on cardiac regeneration and the appropriate frequency of administration of each cell therapy needs to be further investigated [299]. In addition, molecules and/or cells are very often directly injected into the heart during open-surgery. This is an invasive approach that could cause additional injury and pain to the patients. Other less invasive methods, such as intracoronary and intravenous injection, have been employed to deliver cells to the heart. However, these techniques rely on correct homing of cells into the damaged tissue, and very often the delivered cells become trapped in other organs [302,303]. Consequently, other delivery systems that are less aggressive and show the best efficacy and safety need to be developed before CPCs can be applied in regenerative medicine strategies.

There are sufficient reports that support the existence of CPCs within specialized niche structures in the myocardium [241]. For therapeutic applications, these CPCs can be isolated and cultured in vitro, prior to transplantation into the affected heart or, the local microenvironment can be modulated to recruit CPCs to the infarct area. Current biomaterial strategies (discussed in Table 4 and Table 5) have exploited both these methods for functional improvements but do not report complete recovery under physiological conditions or pathological insults. This is evident in the lack of clinical trials with CPCs using biomaterials (Table 6). This offers an opportunity to integrate engineering with mechanistic modulation (perhaps through genetic engineering) to contextualize CPC behavior with disease factors.

The difficulties described above rely, to some extent, on the incomplete understanding of the heart development and cardiac regeneration processes. Increasing this knowledge will clarify the precise stoichiometry of the cardiac factors and optimal culture conditions to accurately mimic the development of CPCs in vitro.

## 10. Final Thoughts—Controversies Surrounding CPCs

It does seem that the debate surrounding CPCs and adult heart repair is taking a full circle—it is there, it is not there, it is there, etc? With the first evidence in rodents supporting the notion of c-KIT^+^ cells from bone marrow or adult heart to replace damaged myocardial tissue, from Piero Anversa’s lab, and subsequent retractions of 31 papers from his group owing to unreliable data, it has encouraged the field to challenge the theory by more robust techniques in mouse models [58,61,304,305]. Results from such studies showed that cardiomyocyte generation from a c-KIT^+^ cells was an extremely rare event. Notably, more recently, the data from Li et al. (2018) showed compelling evidence to support endogenous stem cell to myocyte conversion in embryonic but not in adult heart [306].

Ironically, a more recent work in 2019, by Narino et al., has demonstrated that c-KIT expression labels a heterogeneous cardiac cell population, with cells low in c-KIT expression enriched for CSCs while c-KIT high expressers having endothelial/mast cell differentiation potential [307]. This study went on to show that adult c-KIT-labeled CSCs in mouse “can be myogenic” and help to regenerate after injury and to counteract effects of aging on cardiac structure and function, thus boldly suggesting that CSCs as the bonafide endogenous source of cardiomyocytes in healthy/pathological heart. Consequently, they identified c-KIT haploinsufficiency, generated usually in lineage-tracing studies, prevents efficient labeling of true CSCs on one hand while affecting the regenerative potential of these cells on the other, which perhaps could have been the oversight in the rival camp. Nevertheless, irrespective of the c-KIT controversy, there is no denying that animal studies and clinical trials have appreciated the benefit of a range of cell types for CPCs from many different sources through cellular transplantation approaches ([308,309,310] and Table 1, Table 4 and Table 6). Furthermore, there is an emerging theory that injected/infused CPCs can induce a reconditioning of the injured heart through paracrine signalling or that these cells stimulate an acute inflammatory response when these cells die and are cleared, resulting in a secondary acute healing response [311].

Therefore, as implied in Table 1, there is still no consensus on an endogenous CPC type that is critical for myocardial repair and regeneration but there is growing consensus that regeneration associated with these CPCs are not robust enough to repair severe myocardial damage such as in MI (commented in [307]). While this review does not offer to bias the reader for one or the other theory, in light of these recent studies, it offers the field impetus to interrogate other strategies and CPC sources (like from stem cells or transdifferentiation of somatic cells) to provide mechanistic insights into how CPCs can be more functionally significant in the context of cardiac regenerative medicine.

## 11. Future Directions

Heart failure patients are typically elderly, and suffer from chronic cardiomyopathies and associated complications like diabetes, hypertension, etc. Notably, they possess CPCs with compromised regenerative potential, insufficient to recover lost cardiac function [312,313]. The propensity of CPCs to affect cardiac repair is influenced by several factors, including genetics [314], epigenetic dysregulation [315], environmental stress [315], disease progression and pathogenesis [316,317], heart load [318], medication, and aging [319,320]. Nevertheless, discovery of CPC characteristics has revolutionized the conceptual view of treatment for heart disease, supported by the capacity of CPCs to form functionally integrated cardiomyocytes and vasculature [321]. Therefore, it is rational to enhance potential of CPCs from the adult or reprogrammed cell sources prior to adoptive transfer into a damaged myocardium. Hence, CPC research is gaining momentum to improve its feasibility for cardiac regenerative therapeutics. Advances in this field are progressing towards combining optimised reprogramming approaches from iPSCs and somatic cells with tissue engineering strategies. This will undoubtedly bring advances in genomics, epigenomics, and proteomics of CPCs and their differentiated counterparts, to realise their full potential. Future regenerative approaches might bring together genetic engineering (a very tested strategy in iPSCs), the addition of multiple stimuli (mechanical, electrical and biochemical factors) and tissue engineering approaches to develop a meticulously controlled system that maximises CPC regenerative capacity, and that could potentially be applied in cell therapy, disease modelling, and drug screening (Figure 1).

## Figures and Tables

**Table 1:** Types of CPCs identified in the heart tissue.

CPC Type	Marker Expression	Differential Potential	Functionality of the Differentiated Cells	Applied to Disease In Vivo	Concerns	Ref.
c-KIT	Ki67^+^ NKX2.5^+^ GATA4/5^+^ MEF2C^+^ TBX5^+^ CD45^−^ CD34^−^ CD31^+/−^	-Differentiation trend towards CMs *, ** -Few fibroblasts * -ECs *	In vitro: -Atrial and ventricular CMs and cells of the conduction system * -CMs show a disorganized structure, no sarcomeres, and smaller size than their adult counterparts *, ** In vivo: -CMs couple with host cells and display spontaneous beating and striated structures *, **	-Formation of structural and functional CMs and contribution to coronary vessels in MI rats ** -Reconstitution of a myocardial wall that encompassed up to 70% of LV in MI rats ***	-CPC population is heterogeneous with cells at distinct stage of differentiation and with different commitment to the cardiac lineages *, ** -Differentiated cells show an immature phenotype *, ** -No consensus regarding the regenerative capability of c-KIT CPCs and their lineage marker expression *, ** -Distinct differential potential between neonatal and adult c-KIT^+^ CPCs and between species *, ** -Benefits are mainly a result of paracrine factors *, **	[21,27,28,31,53,58,59,107,108]
SCA1	ISL1^+^ c-KIT^+/−^ PDGFRα^+^ CD105^+^ CD90^+^ CD44^+^ GATA4^+^ MEF2C^+^ NKX2.5^+/−^ TEF-1^+^ CD31^+/−^ CD34^−^ ABCG2^+^	-CMs, SMCs, and ECs *, ** -Foetal SCA1^+^ CPCs tend to differentiate into ECs, whereas adult CPCs have more efficiency towards CMs **	In vitro: -CMs display spontaneous beating, myofilaments and expressed connexin 43 *, ** -Immature CMs and SMCs *, ** -ECs form tube-like structures *, ** -Foetal SCA1^+^ CPCs exhibit more spontaneous beating than adult SCA1^+^ CPCs ** In vivo: -ECs contribute to capillaries and CMs display defined striated structures *	-Knockdown of SCA1 led to larger LV volume, increased infarct rate and limited angiogenesis in MI mice * -SCA1^+^/CD31^−^ cell population numbers increased in the LV following MI * -Transplantation of SCA1^+^/CD31^−^ in MI mice attenuates adverse LV remodeling *	-No human homolog of SCA1 identified ** -SCA1 does not discriminate between proliferating and differentiating cells *, ** -SCA1^+^ CPCs represent a heterogeneous population with subpopulations displaying different lineage potential *, ** -Distinct potency between neonatal and adult SCA1^+^ CPCs ** -Differentiation into CMs requires co-culture with adult/neonatal CMs ** -Benefits are mainly a result of paracrine factors *, **	[11,21,37,64,65,66,67,69,70,109]
KDR/FLK1^low/−^	T^+^ MESP1^+^ c-KIT^−^ GATA4^+^ TBX5^+/^ NKX2.5^+/−^ CD31^+/−^ SL1^+/−^ SMA^+^ PDGFRα^+^	-Highest efficiency for SMCs, followed by CMs and then ECs *, ** -KDR^+^/CXCR4^+^ has better efficiency towards CMs *	In vitro: -CMs display spontaneous Beating *, ** -Predominantly atrial and ventricular CMs ** -Few pacemaker and conduction system cells * -Electrical coupling is observed ** -ECs display LDL-uptake capacity ** -ECs and SMCs form tube-like structures ** In vivo: -Human ESC-derived KDR^+^ CPCs differentiate into CMs and ECs **	-Human ESC-derived KDR^+^ progenitors increased ejection fraction in infarcted hearts of NOD/SCID mice **	-Hematopoietic tendency *, ** -FLK1/KDR marks two populations with distinct cardiac potential that develop at different temporal stages of mesoderm differentiation *	[20,29,79,82,110]
MESP1/2	SSEA1^+^ OCT4^+^ T^+^ KDR^+^ ISL1^+^ TBX5/6/18/20^+^ GATA4/6^+^ NKX2.5^+^ MEF2C^+^ MYOCD^+^ PDGFRα/β^+^ CXCR4^+^ WNT8A^+^ FGF8^+^ HAND2^+^	-More efficiency towards SMCs and ECs *, ** -Some CMs *, **	In vitro: -Formation of ventricular CMs * -CMs express sarcomeric structures when co-cultured with human cardiac fibroblasts and CMs ** In vivo: -CMs display organized myofibrillar striations and express CX43, and SMCs and ECs form tube-like structures and contribute to neovasculogenesis *	-Murine ESC-derived MESP1 CPCs decreased LV-EDV, scar size, and improved LV ejection fraction, stroke volume and cardiac function in MI mice hearts *	-Not fully committed to the cardiac lineages *, ** -Not thoroughly investigated as CPCs *, ** -MESP1 marks a mixed population of CPCs with different multilineage differentiation potential *, ** -MESP1 CPC might be a subset of KDR^+^/PDGFRα^+^ cells *, ** -MESP1 is transiently expressed, making it difficult to track the expansion and differentiation of the CPCs *, **	[72,76,79,80,111]
From First Heart Field (FHF)	NKX2.5^+^ HAND1^+^ TBX5^+^ HCN4^+^	-More efficiency towards CMs *, ** -Some SMCs *, **	In vitro: -Atrial, left ventricle and conduction myocytes *, ** -Presence of both mature and immature CMs * -Some spontaneous beating *, ** -Most CMs display a ventricular-like action potential * -Some atrial-like and nodal-like action potentials are formed * In vivo: -ESC-derived CPCs differentiate into SMCs and CMs, which display beating and form myofibrils *	-Not yet applied in vivo in a disease context	-Difficult to identify and characterized due to lack of markers *, ** -FHF have limited potency *, ** -Not thoroughly investigated as CPCs *, **	[21,84,85,86]
From Second Heart Field (SHF)	ISL1^+^ c-KIT^−/+^ NKX2.5^+/−^ TBX1^+^ GATA4^+^ KDR^+/−^ FGF8/10^+^ FOXH1^+^ MEF2C^+^ WT1^+^	-Majority to CMs, including pacemaker *, ** -Some cardiac fibroblasts, SMCs and ECs *, ** -ISL1^+^/KDR^+^ into ECs and SMCs * -NKX2.5^+^/ISL1^+^ into CMs *, ** -NKX2.5^+^/KDR^+^ into SMCs *	In vitro: -Remarkable contribution to the sino-atrial node * -Only a few towards atrial-ventricular node * -CMs exhibit synchronized calcium transients * In vivo: -Contribution to the coronary arterial system * -SMCs are in the most proximal outflow tract * -ESC-derived ISL1^+^ CPCs differentiate into pacemaker and ventricular CMs, SMCs and ECs * -Knockdown of ISL1 led to a reduction in cardiac tissue formation and affects CPC proliferation, survival and migration *	-Not yet applied in vivo in a disease context	-Majority of contribution to the conduction system is restricted to the sino-atrial node * -EC and SMC contribution is limited to the proximal area of the great vessels * -Embryo-derived SHF show a significant reduction in differentiation into CMs and tripotency was rare *	[22,30,40,83,84,112,113]
Epicardial-derived	WT1^+^ TBX18^+^ SLUG RALDH2 SCA1^+^ PDGFRα^+^	-Vascular SMCs *, ** -CMs under certain in vitro conditions *, ** -Some cardiac fibroblasts (perivascular and interstitial) *, **	In vitro: -SMCs and fibroblasts *,** -Atrial and ventricular CMs, with striated cytoarchitecture, spontaneous contraction, native calcium oscillations and electrical coupling * In vivo: -SMCs contribute to the coronary arteries * -Differentiation into fibroblasts, SMCs and coronary endothelial cells; CMs can be formed when subjected to the stimulation of exogenous factors *	-Epicardial-derived CPCs increased vessel formation and stimulate angiogenesis in murine MI models * -Epicardial-derived CPC conditioned medium reduced infarcted size and improved heart function in MI mice models *,** -Priming of the epicardium with Tβ4 prior to injury led to enhanced migration of epicardial-derived CPCs and generation of CMs in MI mice *	-Epicardial-derived CPCs descend from NKX2.5-and ISL1-expressing cells *, ** -No EC differentiation *, ** -Epicardial-derived CPCs are difficult to culture in Vitro *, ** -No consensus about the level of contribution of the epicardium in cardiac repair *,**	[88,89,90,91,114,115,116,117]
Side Population (SP)	ABCG2^+^ SCA1^+^ CD34^+/−^ CD31^+/−^ c-KIT^−^ NKX2.5^+/−^ GATA4^+/−^ MEF2C^+^ CD45^−^ VE-cadherin^−^	-Fibroblasts & SMCs *, ** -SCA1^+^/CD31^−^ SPs into CMs * -SCA1^+^/CD31^+^ SPs + VEGF into ECs * -CD45^−^ SPs into ECs *	In vitro: -CMs show spontaneous beating and striations on staining * -Electrical coupling is observed when SPs are co-cultured with adult CMs * In vivo: -Differentiation into CMs, forming striated sarcomere structures, SMCs, ECs, and fibroblasts *, ***	-Cardiac SP numbers are significantly increased, particularly in the left ventricle, following acute ischemia ** -Myocardial injury facilitated migration and homing of cardiac SPs *, ***	-Hematopoietic differentiation tendency * -Low percentage of CMs reach advanced maturity *, ** -Contradictory results between different studies on the maturity of the SP-derived CMs *, ** -SPs represent an extremely heterogeneous population * -Complete differentiation requires both cell-intrinsic and -extrinsic factors *	[38,94,96,97,100,101,102,104,118]
Cardiosphere (CS)-derived cells (CDCs)	KDR^+^ c-KIT^+^ SCA1^+^ CD34^+/−^ CD45^−^ CD133^−^ NKX2.5^+^ GATA4^+^ ISL1^+^ CD105^+^/CD31^+^/ CD90^+^/c-KIT^−^ supporting cells	CMs, SMCs & ECs *, **	In vitro: -CMs display spontaneous beating, but lack sarcomeric structure * -Differentiation into ECs and SMCs with VEGF treatment *, ** In vivo: -Differentiation into SMCs and ECs, some potential towards CMs lineages *, ** -Formation of tubular-like structures *	-Transplantation of CDCs/CSs improved cell survival, engraftment and LV ejection fraction, stimulated angiogenesis, inhibited adverse LV remodeling and reduced infarct size in the infarcted mice **	-Human CSs/CDCs require co-culture with adult CMs to stimulate contraction and advance maturity ** -Stemness decreases in monolayer cultures ** -CSs/CDCs represent a mixed cell population *, ** -Benefits result from paracrine factors *, ** -Low CDC engraftment and differentiation efficiency ** -Different markers used, which isolate cells with distinct differentiation potential *, **	[39,106,119,120]

CMs: Cardiomyocytes; SMCs: Smooth Muscle Cells; ECs: Endothelial Cells; MI: Myocardial Infarction; LV: Left Ventricle; EDV: End-Diastolic Volume; LDL: Low Density Lipoprotein; ESC: Embryonic Stem Cell; NOD/SCID: Non-Obese Diabetic/Severe Combined Immunodeficient; VEGF: Vascular Endothelial Growth Factor; *, Mouse; **, Human; ***, Rat.

**Table 2:** Protocols producing CPCs as target cells or as intermediate cells from iPSCs.

Protocol			CPC-Associated Markers Identified	CPCs as Target or Intermediate	Differentiation and Functionality Potential	Limitations	Ref.
Pluripotent Culture	Mesoderm Differentiation	Cardiac Specification					
Mouse iPSCs on feeder-layers and human iPSCs in hESC culture medium without bFGF	Differentiation medium with 20% FBS + gelatin-coated plates + AA between day 2 and 6		NKX2.5^+^ TBX5^+^ & FLK1^+^ CXCR4^+^	Intermediate	-Synchronous beating and better-organized striated myofilaments in CMs	-AA is not able to promote mesodermal differentiation and CM proliferation -No reports on CPC potential into SMCs and ECs	[143]
Human iPSCs in monolayer culture (mTeSR1 + Matrigel-coated plates)	ROCK inhibitor (Y27632) for 1 day and DMEM/F12/B27-vitamin A + BMP4 + AA + CHIR for 3 days		SSEA1^+^ MESP1/2^+^ ISL1^+^	Target	-Differentiation into the three cardiac lineages under specific differentiation media -80% efficiency towards CMs, and 90% into SMCs and ECs -Synchronized beating and presence of organized sarcomeric structures	-Both early and late CPC-related markers were co-expressed in the generated CPCs -Repeated passaging leads to a decrease in CPC proliferation rate -Only one iPSC line was tested	[144]
Human iPSCs on inactivated MEFs followed by feeder depletion culture in Matrigel	BMP4 for 3 days and +/− Activin A + bFGF from day 1 until day 3	DKK1 + VEGF + SB +/− Dorsomorphin/Noggin at day 3	KDR^+^ PDGFRα^+^	Intermediate	-Low yield of CMs (11%)	-iPSC line variability affects protocol’s efficiency and optimal growth factor concentrations -Presence of the CPC population does not always predict efficient differentiation to CMs	[128]
Mouse iPSCs in DMEM with 15% FCS on feeder layers	Differentiation medium with 10% FCS + type IV collagen-coated dishes/OP9 cell sheets for 96–108 h	FLK1^+^ mesodermal cells co-cultured on OP9 cells + differentiation medium + cyclosporin-A	FLK1^+^ CXCR4^+^ VE-cadherin^−^	Target	-Synchronous beating -Pacemaker and ventricular action potentials -Myofilaments formation with transverse Z-bands -Presence of ion channels (Cav3.2, HCN4 and kir2.1) and intercalated disks	-CPCs were only isolated from mouse iPSCs -Differentiation efficiency was different for various iPSC lines -Incomplete human CM maturation	[133]
Human iPSCs on SNL feeder cells and Matrigel-coated plates	Co-culture on END-2 cells + cyclosporin-A at day 8			Target			
Human iPSCs on inactivated MEFs with KO-DMEM medium	Serum-free medium (RPMI/B27) + BMP2 + SU5402 for 6 days		OCT4^+^ SSEA1^+^ MESP1^+^ TBX5^+^ TBX6^+^ TBX18^+^ GATA4^+^ MEF2C^+^ NKX2.5^+^ ISL1^+^ TBX20^+^	Target	-Differentiation towards CMs, SMCs and ECs under specific conditions -Arranged sarcomeric organization and gap junctions when CPCs were co-cultured with either fibroblasts + FCS, cardiac fibroblasts + CMs or conditioned medium -Trend towards ventricular CMs	-Only one iPSC line was tested -SSEA1^+^ CPCs can differentiate into multiple cardiac lineages, like FHF, SHF, epicardium and cardiac neural crest in the presence of FGF signals	[145]
Murine iPSCs on inactivated MEFs	Feeder-free culture on gelatin-coated plates + BIO	IMDM with 15% FCS	FLK1^+^ MESP1^+^ NKX2.5^+^	Target	-Presence of CM, EC and SMC markers	-Incomplete CM maturation -Functionality of the differentiated cells in in vitro conditions needs further assessment	[146]
Human iPSCs on Matrigel-coated plates	E8 medium + ROCK inhibitor for 24 h and RPMI/B27-insulin + CHIR for 48 h/ 4 days		TBX5^+^ NKX2.5^+^ CORIN^+^ HCN4^+^ GATA4^+^	Target	-FHF: mainly differentiates into left ventricular (90%) and some atrial CMs (10%) -Presence of ion channels (Kir2.1) and higher contraction velocity	-4 different CPC populations identified with distinct differentiation potential -Isolation of the CPC populations was performed via a double transgene reporter -Expression of TBX5 and NKX2.5 dynamically changed during differentiation culture, except for the double negative (TBX5^−^/NKX2.5^−^) cell population	[147]
			TBX5^+^ NKX2.5^−^ HCN4^+^ GATA4^+^ WT1^+^ TBX18^+^ KDR^+^ PECAM1^+^	Target	-Epicardial progenitors: contribute to nodal (80%) and some atrial CMs -Formation of tight junctions and expression of the ion channel KCNJ3 -Some potential towards fibroblasts, SMCs and ECs		
			TBX5^−^ NKX2.5^+^ GATA4^+^ MEF2C^+^ ISL1^+^	Target	-SHF: differentiation predominantly into atrial (90%) and some nodal and ventricular CMs -Atrial CMs displayed slower beating rates -Some potential towards SMCs and ECs		
			TBX5^−^ NKX2.5^−^ KDR^+^ PECAM1^+^	Target	-Endothelial potential -Formation of tube-like structures under VEGF		
Human iPSCs on inactivated MEFs followed by EB suspension culture	BMP4 for 4 days	IWR1/IWP1 for 2 days	NKX2.5^+^ ISL1^+^ GATA4^+^ MEF2C^+^	Intermediate	-Low percentage of CMs -Organized sarcomeric structures -Normal calcium transient rhythm	-The CPCs were only identified when using human ESCs -Embryonic action potentials -CPC was an intermediate state during differentiation into CMs	[148]
Human iPSCs on MEFs	DMEM/F12 with 20% FBS + AA + EB plating on gelatin-coated dishes at day 7	MEFs for 24 h and BMP2 + SU5402 for 4 days in RPMI/B27- vitamin A	ISL1^+^ NKX2.5^+^ KDR^+^ MESP1^+^ TXB20^+^ GATA4^+^	Target	-Differentiation towards myocytes and vascular lineages under specific conditions	-Differentiation trend and CM maturation in vitro were not fully assessed	[149]
Human iPSCs on Synthemax-coated plates in E8 medium then mTeSR1/E8 + ROCK inhibitor for 24 h	Albumin-free RPMI + CHIR for 24 h	RPMI + IWP2 for 2 days at day 3 + basal medium at day 5	ISL1^+^ NKX2.5^+^ KDR^+^	Intermediate	-Spontaneous contraction and well-organized sarcomere filaments -Development of ventricular action potentials -Spontaneous calcium transients and connexin 43 expression in CMs	-No information about differentiation potential towards ECs and SMCs	[150]
Human iPSCs on Matrigel in MEF-CM supplemented with bFGF	RPMI/B27-insulin + Activin A for 24 h + BMP4 and bFGF for 4 days	RPMI/B27- insulin + DKK1 for 2 days	MESP1^+^ KDR^+^ ISL1^+^ NKX2.5^+^	Intermediate	-Sarcomere formation -Ventricular and pacemaker action potentials -CM yield varied between 4 and 34%	-Protocol efficiency and CM differentiation and maturation is affected by cell line variability -Incomplete CM maturation -CPC was an intermediate state during differentiation into CMs	[140]
Human iPSCs in Geltrex with E8 medium using spheroid culture	RPMI/B27-insulin + CHIR + BMP4 for 48 h	XAV939 for 48 h at day 4	ISL1^+^ TBX1^+^ FGF10^+^ FGF8^+^ CXCR4^+^ (SHF)	Target	-38% efficiency towards CMs -More potential to generate SMCs, ECs and fibroblasts	-No information about the functionality of the differentiated cells -Only one hiPSC line was tested	[151]
			ISL1^+^ HCN4^+^ TBX5^+^ GATA4^+^ CXCR4^−^ (FHF)	Target	-62% efficiency towards CMs -Low levels of EC and fibroblast markers		
Human PSCs on Matrigel/Synthemax-coated plates in mTeSR1/E8 medium with ROCK inhibitor	CHIR in RPMI basal medium for 24 h	IWP2/IWP4 in RPMI basal medium from day 3 to day 5 + LaSR basal or RPMI/Vc/Ins with ROCK inhibitor at day 6 + CHIR for 48 h from day 7	WT1^+^ TBX18^+^ TCF21^+^ ALDH1A2^−^ KDR^+^	Target	-Differentiation towards fibroblasts and SMCs -Fibroblasts and SMCs display fibroid spindle-like shape and a fusiform appearance, respectively -Formation of mature epithelial-like sheets with tight junctions (cobblestone morphology and expression of ZO1 along cell borders) -SMCs display calcium transients and contractibility	-Epicardial progenitor cells are derived from a more multipotent CPC population (PDGFRα^+^/ ISL1^+^/NKX2.5^+^/GATA4^+^/TBX5^+^) -Format size of the culture (i.e., 96-well or 6-well plate) affects maturity of the epicardial cells -Different protocols lead to the formation of mesodermal cells expressing distinct markers (PDGFRα^+^/KDR^+^ and ISL1^+^/NKX2.5^+^) -Epicardial progenitor cells exhibit multiple origins	[152]
	Albumin-free RPMI + CHIR for 24 h	RPMI + IWP2 for 2 days at day 3 + RPMI/Vc/Ins with ROCK inhibitor for 24 h at day 6 + CHIR in RPMI//Vc/Ins for 48 h at day 7		Target			[153]
Human iPSCs on inactivated MEFs	StemPro-34 medium + BMP4 for 24 h + BMP4, Activin A and bFGF from day 1 until day 3	StemPro-34 medium + Matrigel-coated plates + BMP4 + CHIR + SB + VEGF for 2 days		Target			[154]
Human iPSCs in CDM + BSA + Activin A + FGF2 on gelatin-coated plates	CDM + PVA + FGF2 + LY294002 + BMP4 for 36 h and CDM + PVA + FGF2 + BMP4 for 3.5 days	CDM + PVA + BMP4 + WNT3A + RA for 10 days		Target			[155]
Human iPSCs in E8 medium and monolayer culture on vitronectin-coated plates	S12-insulin medium + CHIR for 24 h	S12-insulin medium + IWR1 for 48 h at day 3 and RA + CHIR between day 5 and 8		Target			[156]
Murine iPSCs in inactivated MEFs in SCM	SCM-LIF + AA at day 2	Puromycin at day 6 for 3 days	NKX2.5^+^ c-KIT^+^ FLK1^+^ SCA1^+^	Target	-Differentiation potential towards ventricular CMs, SMCs and ECs -Sarcomeric organization and intracellular coupling observed	-Presence of CPCs expressing different sets of markers -Application of a plasmid system for CPC enrichment	[157]
Human iPSCs on MEFs followed by suspension culture in ESC culture medium	Gelatin-or human laminin211-coated plates + IMDM-serum and CHIR + BIO for 3 days	KY02111 +/− XAV939 or IWP2 from day 3 until day 9	NKX2.5^+^ GATA4^+^	Intermediate	-Predominantly ventricular CMs and 16% pacemaker cells -Spontaneous beating, sarcomere myofilaments, Z-bands, ion channels (HERG and KCNQ1) intercalated disks observed	-Mechanism of canonical WNT inhibition by KY02111 not fully understood -Protocol efficiency is affected by the presence of serum and cytokines -No differentiation into SMCs and ECs	[131]
Human iPSCs in E8 medium on Synthemax/Matrigel-coated plates	CDM3 medium (RPMI basal medium + AA + rice-derived RHA) + CHIR for 2 days	CDM3 medium + WNT-C59 for 48 h at day 2	MESP1^+^ KDR^+^ ISL1^+^ GATA4^+^ NKX2.5^+^ TBX5^+^ MEF2C^+^	Intermediate	-Formation of atrial, ventricular and nodal CMs	-Presence of unspecified CMs, without a defined subtype -Incomplete CM maturation -No differentiation into SMCs and ECs -CPC was an intermediate state during differentiation into CMs	[137]
Human iPSCs in mTeSR1 + ROCK inhibitor on Matrigel/Synthemax	Pre-treatment with CHIR/BIO for 3 days	RPMI/B27-insulin + Activin A for 24 h + BMP4 for 4 days	ISL1^+^ NKX2.5^+^	Intermediate	-High yield of CMs -Normal sarcomere organization with transverse Z-bands -Presence of intercalated disks -Maturation trend towards ventricular CMs (80–90%) Some atrial-like action potential (10%) and absence of nodal-like potentials -Some formation of SMCs	-Optimal BMP4 concentration varies with different cell lines -Heterogenous activation of the canonical WNT signaling upon CHIR treatment in transgenic iPSC lines -Requirement of long periods of time (>60 days) to reach advanced CM maturity -Greater efficiency observed in studies using transgenic models	[126,130]
Transgenic iPSC lines carrying lentiviral integrated β-catenin shRNA	CHIR in RPMI/B27-insulin for 24 h	Doxycycline at 36 h post-CHIR addition	ISL1^+^ NKX2.5^+^ TBX5^+^ WT1^+^	Intermediate			
Non-transgenic hiPSC lines		IWP4 or IWP2 at day 3	Not reported	-			[130]
		IWP2 at day 3	ISL1^+^ NKX2.5^+^	Intermediate			[126]
Human iPSCs on vitronectin-coated plates in mTeSR1 + ROCK inhibitor for 24 h	RPMI/B27-insulin + ISX-9 for 7 days		NKX2.5^+^ GATA4^+^ ISL1^+^ MEF2C^+^	Target	-Differentiation potential towards CMs, ECs, and SMCs in vitro and in vivo -CMs displayed myofilaments, mitochondria and glycogen particles -Formation of tube-like structures and LDL-uptake in ECs -ECs, and SMCs formed vascular structures in vivo	-The exact mechanisms by which ISX-9 induces the expression of cardiac transcription factors is unclear -No reports about electric coupling between generated CMs and endogenous CMs in vivo -No information about the electrophysiology of CMs	[136]
Human iPSCs on Matrigel in mTeSR1 + ROCK inhibitor	CHIR in RPMI/B27-insulin for 24 h + bFGF	IWP2 from day 3 to day 5	MESP1^+^ T^+^ GATA4^+^ ISL1^+^ NKX2.5^+^ TBX1^+^ HAND2^+^ at day 2–3 & KDR^+^ PDGFRα^+^ at day 4–5	Intermediate	-Formation of SHF-derived CPCs -Differentiation trend into fibroblasts, which exhibited characteristics of fetal ventricular fibroblasts	-Stage-specific progenitors were generated with this protocol -Differentiation potential was limited to fibroblasts -The fibroblasts generated might represent just one of the populations of cardiac fibroblasts present in the native heart -Only one hiPSC line was tested (line variability effects need further assessment)	[158]
Human iPSCs in feeder-free (Geltrex) monolayer culture	RPMI + PVA + BMP4 + FGF2 for 2 days	RPMI-insulin + 20% FBS/human serum for 2 days	MESP1^+^ ISL1^+^ NKX2.5^+^	Intermediate	-Robust contraction -Striated sarcomeres and gap junction formation -High yield of CMs (64–89%) -Presence of physiological calcium transients and functional electrical coupling -Differentiation trend into ventricular CMs	-FBS is undefined -Incomplete CM maturation -CPC was an intermediate state during differentiation into CMs	[122]
		RPMI-insulin + 20% HSA + AA for 2 days		Intermediate			
		RPMI-insulin + 20% HSA + AA for 2 days		Intermediate			

hiPSCs: human iPSCs; (h)ESC(s): (human) Embryonic Stem Cell(s); b(FGF): (basic) Fibroblast Growth Factor; FBS: Foetal Bovine Serum; AA: Ascorbic Acid; CM(s): Cardiomyocyte(s); SMC(s): Smooth Muscle Cell(s); EC(s): Endothelial Cell(s); DMEM/F12/B27: Dulbecco’s Modified Eagle Medium/Ham’s F12 Nutrient Mixture/B27 serum supplement; BMP: Bone Morphogenic Protein; CHIR: CHIR99021; MEF(s): Murine Embryonic Fibroblast(s); DKK1: Dickkopf WNT signaling Pathway Inhibitor 1; VEGF: Vascular Endothelial Growth Factor; SB: SB-431542; FCS: Foetal Calf Serum; OP9: Mouse bone marrow-derived stromal cells; SNL: Mouse Fibroblast STO cell line-derived feeder cells; END-2: Visceral Endodermal Stromal cells; KO-DMEM: KnockOut DMEM; RPMI/B27: Roswell Park Memorial Institute/B27; FHF: First Heart Field; SHF: Second Heart Field; BIO: 6-bromoindirubin-3′-oxime; IMDM: Iscove’s Modified Dulbecco’s Medium; EB: Embryoid Body; IWR: Inhibitor of WNT Response; IWP: Inhibitor of WNT Production; MEF-CM: MEF-Conditioned Medium; LaSR: advanced DMEM/F12 with ascorbic acid; RPMI/Vc/Ins: RPMI with Ascorbic Acid (Vc) and Insulin (Ins); ZO1: Zonula Occludens-1/Tight junction protein-1; CDM: Chemically Defined Medium; BSA: Bovine Serum Albumin; PVA: Polyvinyl Alcohol; RA: Retinoic Acid; S12: Chemically Defined S12 Differentiation Medium; SCM: Stem Cell Medium; LIF: Leukaemia Inhibitor Factor; RHA: Recombinant Human Albumin; shRNA: small hairpin RNA; ISX-9: isoxazole; HSA: Human Serum Albumin.

**Table 3:** Role of microRNAs in CPC biology.

CPC Property		MiRNA Involved	Target Protein/Pathway	Mechanism	Ref.
Proliferation		miR-21	PTEN	Inhibit negative regulators of cell proliferation	[224]
		miR-218	SFRP2		
		miR-548c	MEIS1		
		miR-509			
		miR-23b			
		miR-204	ATF2	Repress proliferation-related transcription factors and induces differentiation	[225]
		miR-1	HDAC4		
			HAND2		
		miR-200b	GATA4		
		miR-17-92 cluster	Not reported	Increases proliferation rate	[219]
Differentiation	CMs	miR-133	NELFA	Suppresses cardiogenesis	[226]
		miR-218	SFRP2	Inhibits a negative regulator of cell proliferation	[227]
		miR-142	MEF2C	Suppresses CM formation	[228]
		miR-1	DLL1	Increases NKX2.5 and Myogenin expression	[229]
		miR-499	ROD1	Suppresses inhibitory factors of cardiac differentiation	[224,230]
			SOX6		
		miR-708	N-RAS		[231]
		miR-322-503 cluster	CELF1		[220]
	SMCs	miR-22	EVI1	Inhibits negative regulators of SMC marker gene expression and of SMC transcription factors	[232]
		miR-29a	YY1		[233]
	miR-669a		MYOD	Increases CPC differentiation potential by preventing skeletal myogenesis	[234]
	miR-669q				
Migration	miR-206		TIMP3	Suppresses a metalloproteinase inhibitor	[235]
	miR-21		PTEN	Promotes migration of SCA1^+^ CPCs (not fully clear)	[236]
Apoptosis	miR-21		BIM	Inhibit apoptotic activators	[237]
			PDCD4		
	miR-24		BIM		
	miR-221				
Necrotic Cell Death	miR-155		RIP1	Inhibits necrosis activators	[238]
Vascular Remodeling	miR-221		c-KIT eNOS	Inhibit endothelial cell migration and proliferation	[239]
	miR-222				
Cell Repolarization	miR-1		KCNE1 KCNQ1	Reduce potassium current in hyperglycemia conditions	[240]
	miR-133				

CM(s): Cardiomyocyte(s); SMC(s): Smooth Muscle Cell(s); PTEN: Phosphatase and Tensin Homolog; SFRP2: secreted Frizzled-Related Protein 2; MEIS1: Meis Homeobox 1; ATF2: Activating Transcription Factor 2; HDAC4: Histone Deacetylase 4; NELFA: Negative Elongation Factor-A; DLL1: Delta-Like protein 1; ROD1: Regulator of Differentiation 1; N-RAS: Neuroblastoma RAS Viral Oncogene Homolog; CELF1: CUG-binding Protein Elav-like Family Member 1; EVI1: Ecotropic Virus Integration Site 1 Protein Homolog; YY1: Transcription Factor Yin Yang 1; MYOD: Myoblast Determination Protein 1; TIMP3: Tissue Inhibitor of Metalloproteinase 3; BIM: BCL2-like Protein 11; PDCD4: Programmed Cell Death 4; RIP1: Receptor-Interacting Protein Kinase 1; eNOS: endothelial Nitric Oxide Synthase; KCN-E1/-Q1: Potassium Voltage-Gated Channel Subfamily E Member 1/Subfamily Q Member 1.

**Table 4:** Cardiac tissue engineering strategies with biomaterials using CPCs.

Scaffold Biomaterial	Experimental Design	Outcome	Limitations	Ref.
Fibrin patch	SSEA1^+^ and ISL1^+^ hESCs-CPCs mixed in fibrinogen, and scaffolds were then transplanted into myocardial infarction rats	-Improved contractility and decrease in adverse ventricular remodeling -Increased angiogenesis and attenuation of fibrosis	-Poor long-term cell engraftment -Functional improvements resulted from paracrine signaling	[247]
	Same process as above, except the scaffolds were delivered surgically on the infarct area of a 68-year-old patient suffering from severe heart failure	-No observation of ventricular arrhythmias -Decreased in adverse ventricular remodeling	-Presence of T-cell response 3 months post-implantation -Absence of neovascularization in patch-treated area	[248]
	mESCs were primed with BMP2 for 36 h and seeded into fibrin matrices The constructs were then implanted onto normal or infarcted rat left ventricles	-Efficient cell engraftment -Attenuation of left ventricle dilation -Promotion of neovascularization	-Rapid inflammation-driven degradation of scaffolds -Unclear whether neovascularization was due to in situ cell differentiation or endogenous EC recruitment	[249]
Polyethylene glycol diacrylate woodpile (PEGDa-Wp) and PEGDa hydrogel.	Human adult LIN^−^/SCA1^+^ CPCs were seeded in a PEGDa hydrogel and the mixture was then cultured onto a PEGDa-Wp	-Benefits on cell assembly and alignment -Induction of cell spatial-ordered multilayer organization and differentiation towards a CM phenotype	-Incomplete maturation of CMs -No differentiation into SMCs and ECs -No in vivo testing of the scaffolds	[258]
Poly(l-lactic acid) Nanofibres	mESC-derived ISL1^+^/GATA4^+^ CPCs were seeded onto nanofibres After 7 days of in vitro differentiation, the scaffolds were implanted subcutaneously in the dorsal area of athymic nude mice	-Enhancement of cell attachment, extension and differentiation in vitro -Improvement of cell survival, integration and commitment to the three cardiac lineages in vivo -Induction of angiogenesis in vivo	-Poor in vitro differentiation into ECs -Unclear whether neovascularization was due to paracrine factors or CPC-derived SMCs and ECs	[257]
Tissue Printing using Sodium Alginate	Human SCA1^+^ CPCs were mixed with alginate matrixes, including an RGD-modified alginate, which were then used to print porous and non-porous scaffolds	-Porosity preserved viability and proliferation and increased cardiac commitment of CPCs -CPCs migrated from the construct and formed tubular-like structures	-Incomplete maturation of the differentiated cells -No in vivo testing of the scaffolds	[250]
Porcine- and human-derived myocardial matrices	Human SCA1^+^ CPCs were seeded onto porcine and human ECM Scaffolds were injected into the left ventricular free wall of healthy hearts of Sprague Dawley rats	-Porcine-derived ECM was more efficient at promoting CPC differentiation, whereas human-derived ECM promoted CPC proliferation	-Variation in ECM properties due to distinct decellularised methods used, patient-to-patient variability and tissue age	[259]
3D-printed hyaluronic acid/gelatin-based matrix	Human SCA1^+^ CPCs were printed together with the matrix The cell-loaded patches were transplanted in myocardial infarction mice	-Reduction of adverse remodeling and fibrosis -Long-term CPC survival and engraftment -Formation of vessel-like structures within the scaffold in vivo	-Absence of neovascularization in the infarcted region -Incomplete maturation of CMs in vivo	[251]
Collagen/Matrigel hydrogels	Human SCA1^+^ CPCs were encapsulated in collagen/Matrigel hydrogels which were cultured in either stress-free or unidirectional constrained conditions	-Enhanced cardiac differentiation and matrix remodeling -Constrained hydrogels stabilized CPC viability, attachment and proliferation -Static strain stimulated actin fiber formation and cell alignment	-Differentiation trend towards CMs -Incomplete maturation of CMs -No CPC differentiation into SMCs and ECs -No in vivo testing	[260]
Decellularised porcine ventricular ECM	Human Foetal and adult SCA1^+^ CPCs were resuspended in porcine myocardial matrix and collagen type I solutions The cell/matrix mixtures were injected into the left ventricular wall of Sprague Dawley rats	-The myocardial matrix improved CPCs adhesion, survival, proliferation and cardiac commitment both in vitro and in vivo -Foetal CPCs survived better than adult CPCs in vivo	-Rats were euthanized 30 min post-implantation, preventing assessment of long-term effects on cell survival, migration and cardiac function	[261]
	Same procedure as above, exceptions: use of adult rat c-KIT^+^ CPCs and no in vivo implantation	-The cardiac ECM improved cardiac commitment, cell survival, proliferation and adhesion	-Differentiation trend towards CMs. -Low differentiation efficiency towards ECs and SMCs	[262]
Whole decellularised mouse heart	hiPSC- and hESC-derived KDR^+^/c-KIT^−^ CPCs were seeded into a whole decellularised mouse heart The repopulated hearts were perfused with VEGF and DKK1 or VEGF and bFGF	-Efficient control of in situ iPSC-CPC differentiation -Advanced CM maturation -Development of vessel-like structures and spontaneous contraction for both iPSC-and ESC-CPC constructs	-Scattered regions of uncoupled cells -Insufficient mechanical force generation and incomplete electrical synchronization of the constructs	[252]
	FLT1 (VEGFR1)^+^/PDGFRα^+^ hESC-CPCs were seeded onto decellularised mice hearts, which were implanted subcutaneously into SCID mice	-In situ generation of CMs, SMCs and ECs -Formation of a vascular network and higher expression of CM markers in vivo	-In vivo differentiated ECs were not ubiquitously distributed in the decellularised scaffold -Absence of beating populations	[263]
Whole decellularised rat heart	hESC-derived KDR^+^/PDGFRα^+^ CPCs were expanded in a stirred-suspension bioreactor and seeded onto perfusion-decellularised *Wistar* rat hearts containing immobilized bFGF	-Improved CPC retention, proliferation and cardiac differentiation potential -Spontaneous and synchronous contractions -Advanced CM maturation	-Growth factor immobilization prevents spatiotemporal control -No in vivo testing	[264]
Whole decellularised human heart	Human adult c-KIT^+^ CPCs from human cardiac biopsies were cultured onto perfused-decellularised heart ventricles	-Increased CPC growth and stimulated differentiation towards cardiac lineages in vitro	-Poor CPC infiltration into the matrix -No electrical signal propagation. -No in vivo testing	[265]
Rat and pig collagen matrix and decellularised left ventricle ECM	iPSC-CPCs were cultured on rat or pig collagen matrices and decellularised ECM CPCs were also co-cultured with ECs and CMs	-Enhanced expression of contractile protein gene expression -Cell communication was observed in co-cultures	-No results reported on CPC proliferation and differentiation -No information about the CPC markers	[253]
3D-bioprinted patch containing decellularised porcine ventricular ECM	Bioinks composed of decellularised ECM, human neonatal c-KIT^+^ CPCs and gelatin methacrylate were used to print patches, which were implanted onto the epicardial surface of the right ventricle of Sprague Dawley rat hearts	-Good CPC retention and viability in the scaffolds -Enhanced cardiogenic differentiation and angiogenic potential -Presence of vascularization in the patches in vivo	-Main purpose of the patch was to improve the paracrine release from the CPCs -No influence in SMC differentiation	[266]
Foetal and adult rat decellularised ventricle ECM	Immortalized adult mouse LIN^−^/SCA1^+^ CPCs were seeded onto embryonic, neonatal and adult rat ECM	-Good CPC retention, motility and viability -Remodeling of the supporting ECM -Enhanced production of cardiac repair factors	-No evidence of CPC differentiation -No in vivo testing	[267]
Decellularised murine embryonic heart	Day 5 and 9 mESC-CPCs were then seeded onto the decellularised scaffolds	-Day 5 progenitors formed spontaneously beating constructs in the scaffolds	-Mixed cell population isolated -Not all cell populations led to functional maturation	[268]
Decellularised human pericardium-derived microporous scaffold	Human SCA1^+^ CPCs were seeded onto 3D microporous pericardium scaffolds, which were then implanted subcutaneously into Wistar rats	-Improved CPC migration, survival, proliferation and differentiation -Reduction of immunological response and enhanced angiogenesis	-No influence in CPC differentiation towards SMCs	[269]
Self-assembling peptide nanofibers	Adult LIN^−^/c-KIT^+^ rat CPCs were seeded onto IGF1-tethered nanofibres CPCs and scaffolds were injected into myocardial infarction rats	-Enhanced CPC survival, proliferation and differentiation into CMs -Improved angiogenesis, recruitment of resident CPCs and attenuation of ventricle dilation	-Growth factor immobilization prevents spatiotemporal control -Newly formed CMs were derived from resident CPCs -CPCs were not cultured on the scaffolds prior to implantation	[254]
	Adult mouse SCA1^+^ CPCs were mixed with Puramatrix^®^ complex and injected into the border area of the myocardium in myocardial infarction mice	-Reduction of the infarct area and attenuation of ventricular dilation. -Enhanced neovascularization	-No CPC differentiation towards ECs -Functional improvements resulted from paracrine signaling -Poor CPC engraftment	[255]
RDG-modified collagen and porous gelatin solid foam	Human adult CS-CPC were grown as secondary CSs, which were seeded onto the scaffolds	-Enhanced cell migration and ECM production -Increased CPC cardiogenic potential, cell retention and adherence	-Cardiac commitment trend towards CMs -Distinct scaffold morphologies promoted different biological processes	[270]
Degradable Poly(*N*-isopropylacrylamide) hydrogel	Mouse CDCs were added into hydrogel solutions, with or without collagen and containing different stiffness	-Preservation of CDC proliferation -Stimulation of differentiation into mature cardiac cells in hydrogels with medium stiffness and collagen	-No differentiation into ECs and SMCs -No in vivo testing	[256]
Biodegradable gelatin	Human CDCs were seeded onto bFGF immobilized gelatin hydrogels, which were implanted in the epicardium of immunosuppressed myocardial infarction pigs	-Enhanced angiogenesis, cell engraftment -Reduction of the infarct area and attenuation of adverse ventricular remodeling	-Growth factor immobilization prevents spatiotemporal control -No differentiation into ECs and SMCs	[271]
Fibrinogen/Matrigel mixture and PDMS molds	NKX2.5^+^/c-KIT^+^/either FLK1^+^ or SCA1^+^ iPSC-CPCs were mixed in a fibrinogen/Matrigel hydrogel and applied into PDMS molds	-Spontaneous and synchronous contraction -Highly organized sarcomere structures and robust electromechanical connections	-Improper nutrient access within the construct -No differentiation potential towards SMCs and ECs -No in vivo testing	[157]
Collagen sponge	CPCs were seeded onto collagen sponges and then transplanted into rat hearts with atrioventricular conduction block	-Enhanced vascularization -Gap junction formation -Differentiation into CMs, conduction cells and ECs	-No information about the functionality of the CPC-derived cells	[272]

(h/m)ESC(s): (human/murine) Embryonic Stem Cell(s); BMP: Bone Morphogenic Protein; EC(s): Endothelial Cell(s); CM(s): Cardiomyocyte(s); SMC(s): Smooth Muscle Cell(s); ECM: Extracellular Matrix; VEGF: Vascular Endothelial Growth Factor; DKK1: Dickkopf WNT Signaling Pathway Inhibitor 1; bFGF: basic Fibroblast Growth Factor; hiPSC(s): human induced Pluripotent Stem Cell(s); SCID: Severe Combined Immunodeficiency; IGF1: Insulin-like Growth Factor; CS(s): Cardiosphere(s); CDC(s): Cardiosphere-Derived Cell(s); PDMS: polydimethylsiloxane.

**Table 5:** In vitro cardiac tissue engineering techniques with biomaterials to stimulate and record hPSC-CM electrical activity.

Cells	Biomaterial/Scaffold	Platform	Stimulation	Electrophysiology	Ref.
hiPSC-CMs	Graphene substrate	2D	FET (current pulse with f = 1 Hz) For calcium: voltage ramp from −80 to +60 mV at 20 mV/s	-Enhanced electrophysiological properties: RP = −40.54 ± 1.72 mV AP = 75.24 ± 3.91 mV CV = 5.34 ± 1.60 cm/s I_Ca2+_ density = −9.31 ± 2.35 pA/pF I_Ca2+,L_ density = −2.47 ± 0.6 pA/pF I_k_ density = 46.24 ± 8.45 pA/pF I_kr_ density = 36.57 ± 5.84 pA/pF Ca^2+^ transients: Amplitude intensity = 1.69 ± 0.20 u Upstroke velocity 3.09 ± 0.99 u/s Decay velocity (50%) = 0.84 ± 0.29 s	[273]
iCell^®^ CMs & hESC-CMs	Reduced graphene oxide (rGO)	2D	Light: intensity >1 mW/mm^2^, duration 40-ms-2-Hz light pulses and 3-s step of light	-Optical stimulation on rGO substrates improves CMs electrophysiology -rGO increases AP peaks frequency -On rGO CMs contraction frequency increases with light intensity	[274]
Neonatal Sprague Dawley rat vCMs	Electrospun gelatine + PCL nanofibres	3D	FET (1–3 V, 50-ms-long pulses at 1–2 Hz)	-Electrical stimulation results in regularly spaced spikes (f = 1–2 Hz) with shape and width consistent with CM extracellular signals -NE increases electrical activity and frequency of calcium transients	[275]
hiPSC-CMs	PLGA electrospun aligned nanofibres	3D	Not applied	-Enhanced CM maturity and electrical activity -CM drug (E4031) response showed higher electrophysiological homogeneity -L-ANFs increased FP amplitude, number of electrically active cells, synchronization and anisotropic propagation of the electrical signal	[276]
hESC-CMs & hiPSC-CMs	Type I collagen gel template suture (Biowires)	3D	Electrical field with daily and progressively frequency increase: low frequency ramp-up regimen (from 1 to 3 Hz) or high frequency ramp-up regimen (from 1 to 6 Hz)	-Electrical stimulation enhanced electrical activity frequency -High frequency increased electrophysiological properties, contractile activity, synchronization and CV -High frequency decreased excitation threshold and variability in AP duration -High frequency improved CM response to caffeine and Ca^2+^ handling properties: I_ERG_ = 0.81 ± 0.09 pA/pF I_K1_ = 1.53 ± 0.25 pA/pF	[277]
hESC-CMs	MEA coated with collagen type I + agarose layer	2D	Anti-arrhythmic and pro-arrhythmic drugs	-Pharmacological stimulation influences CMs electrophysiology -FPD and CT are dependent on the dose of arrhythmogenic drugs: E-4031 & Astemizole increased FPD Flecainide & Terfenadine decreased FPD Flecainide, Astemizole & Terfenadine increased CT and of safe drugs: Verapamil & Lidocine decreased FPD Lidocine slightly increased CT	[278]
hiPSC-CMs	MEA coated with hydrogel containing fluorescence microbeads	2D	Electrical: periodic voltage pulses (biphasic square waves with pulse width = 4 ms, f = 0.2 Hz, peak-to-peak amplitude = 4 V) Pharmacological: drug exposure (NE and Blebbistatin)	-Good electrical coupling of CMs (FP = 9–35 µV and CV = 16 cm/s) -Electrical pacing promoted synchronized contraction (f = 11 bpm) -Recorded impedance increased with cell attachment and at each contraction -Blebbistan inhibited beating activity and has no effect on FP -NE increased CV and contraction spikes rate	[279]

hiPSC(s): human induced Pluripotent Stem Cell(s); hESC(s): human Embryonic Stem Cell(s); (v)CM(s): (ventricular) Cardiomyocyte(s); FET: Field Effect Transistor; f: frequency; RP: Resting Potential; AP: Action Potential; CV: Conduction Velocity; PCL: Polycaprolactone; NE: Norepinephrine; PLGA: Poly(lactic-co-glycolic) acid; L-ANFs: Low-density nanofibres; FP: Field Potential; FPD: Field Potential Duration; CT: Condition Time; MEA: Micro-Electrode Array; IDE: Interdigitated electrode.

**Table 6:** Past and ongoing clinical trials using CPCs.

Clinical Trial Name	Phase	Start/End Date	CPC Type	Delivery of Cells	Biomaterial Added	Results	Ref.
**CADUCEUS** prospective, randomized trial	I	2009–2012	CDCs	Direct injection via catheter	none	LVEF unchanged at 12 months Scar size decreased 12.3% at 12 months Regional contractility and systolic wall thickening increased	[283,293]
**ALCADIA** Open-label, non-randomized trial	I	2010–2013	CDCs	Direct injection via catheter	Biodegradable gelatin hydrogel sheet containing 200 μg of bFGF planted onto epicardium covering the injection site	LVEF increase 12% at 6 months Scar size decrease 3.3% at 6 months	[285]
**ALLSTAR** Open-label cohort (PI), double-blinded, randomized, placebo-controlled study (PII)	I/II	2012–2019	CDCs	Direct injection via catheter	none	Terminated (follow-up activities were ceased)	[294]
**ESCORT** Open-label trial	I	2013–2018	ESC-derived ISL1^+^/CD15^+^	Epicardial patch via coronary artery bypass procedure	Fibrin gel patch containing progenitor cells	LVEF increase of 12.5% No arrhythmias, or tumor formation	[287]
**CAREMI** Double blinded, randomized, placebo-controlled trial	I/II	2014–2016	CDCs	Direct injection via catheter	none	Infarct size decreased to 15.6% at 12 months LVEF increase of 7.7% at 12 months	[295]
**DYNAMIC** Open-label trial, randomized, double-blinded, placebo-controlled trial	I	2014–ongoing	CDCs	Direct injection via catheter to multi-vessel areas of heart	none	Ongoing	[296]
**CONCERT-HF** Randomized, double-blinded, placebo-controlled trial	II	2015–ongoing	c-KIT^+^	Direct injection via catheter	none	Ongoing (paused on 29.10.18, re-approved 06.02.2019)	[297]
**TICAP** Open-label trial, non-randomized	I	2011–2013	CDCs	Direct injection via catheter	none	RVEF increase of around 8.0% at 18 and 36 months No tumor formation	[288,290]
**PERSEUS** Open-label trial, randomized	II	2013–2016	CDCs	Direct injection via catheter	none	LVEF increase of 6.4% at 3 months Reduction in scar size	[289]
**APOLLON** Randomized, single-blinded	III	2016 & Unknown	CDCs	Direct injection via catheter	none	Unknown status (last update was September 2017)	[291]
**TICAP-DCM** Randomized	I	2017–ongoing	CDCs	Direct injection via catheter	none	Recruiting	[292]
**REGRESS-HFpEF** Randomized, double-blinded, placebo-controlled trial	II	2017–ongoing	CDCs	Direct injection via catheter	none	Ongoing	[298]

CDCs: Cardiosphere-Derived Cells; ESC: Embryonic Stem Cell; bFGF: basic Fibroblast Growth Factor; LVEF: Left Ventricular Ejection Fraction; RVEF: Right Ventricular Ejection Fraction.
